# Fungal species and mycotoxins in mouldy spots of grass and maize silages in Austria

**DOI:** 10.1007/s12550-022-00453-3

**Published:** 2022-03-26

**Authors:** Felipe Penagos-Tabares, Ratchaneewan Khiaosa-ard, Marlene Schmidt, Cátia Pacífico, Johannes Faas, Timothy Jenkins, Veronika Nagl, Michael Sulyok, Roman Labuda, Qendrim Zebeli

**Affiliations:** 1 Institute of Animal Nutrition and Functional Plant Compounds, University of Veterinary Medicine Vienna, Veterinaerplatz 1, 1210 Vienna, Austria; 2 BIOMIN Research Center, Technopark 1, 3430 Tulln a.d. Donau, Austria; 3 Department IFA-Tulln, University of Natural Resources and Life Sciences (BOKU), Konrad Lorenzstrasse 20, 3430 Tulln, Austria; 4 Institute of Food Safety, Food Technology and Veterinary Public Health, University of Veterinary Medicine Vienna, Veterinaerplatz 1, 1210 Vienna, Austria; 5 Research Platform Bioactive Microbial Metabolites (BiMM), 3430 Tulln a.d. Donau, Austria; 6 Department for Farm Animals and Veterinary Public Health, Christian-Doppler-Laboratory for Innovative Gut Health Concepts in Livestock (CDL-LiveGUT), University of Veterinary Medicine Vienna, Veterinaerplatz 1, 1210 Vienna, Austria

**Keywords:** Silage quality, Spoilage, Fungal contamination, Multi-mycotoxin analysis, Dairy farm

## Abstract

Fungi and mycotoxins in silage can have detrimental consequences for both cattle and human health. This pilot study identified, via the routinary direct plating method, the dominant cultivable fungi in mouldy grass silages (GS) (*n* = 19) and maize silages (MS) (*n* = 28) from Austria. The profiles of regulated, modified, and emerging mycotoxins together with other fungal metabolites were analysed via LC-(ESI)MS/MS. *Penicillium roqueforti*, *Saccharomyces* spp., *Geotrichum candidum*, *Aspergillus fumigatus* and *Monascus ruber* were the most frequent fungal organisms identified. Other species including *Mucor circinelloides, Fusarium* spp. and *Paecilomyces niveus* were detected at lower frequencies. The presence of complex mixtures of toxic and potentially toxic compounds was evidenced by high levels and occurrences (≥ 50%) of *Penicillium*-produced compounds such as mycophenolic acid (MPA), roquefortines (ROCs), andrastins (ANDs) and marcfortine A. Mouldy silages contained toxins commonly produced by genus *Fusarium* (e.g. zearalenone (ZEN) and trichothecenes), *Alternaria* (like tenuazonic acid (TeA) and alternariol (AHO)) and *Aspergillus* (such as sterigmatocystin (STC)). Compared to those in GS, mouldy spots in MS presented significantly higher fungal counts and more diverse toxin profiles, in addition to superior levels of *Fusarium* spp., *Penicillium* spp. and total fungal metabolites. Generally, no correlation between mould counts and corresponding metabolites was detected, except for the counts of *P. roqueforti*, which were positively correlated with *Penicillium* spp. metabolites in mouldy MS. This study represents a first assessment of the fungal diversity in mouldy silage in Austria and highlights its potential role as a substantial contributor to contamination with complex mycotoxin mixtures in cattle diets.

**Supplementary information:**

The online version contains supplementary material available at 10.1007/s12550-022-00453-3.

## Introduction

Silage production is a widespread practice applied to preserve the nutritional value of forages for livestock feeding, using spontaneous lactic fermentation under anaerobic conditions (Muck et al. 2018). Grass silage (GS) and (whole plant) maize silage (MS) are the most frequently used dietary ingredients in modern dairy and beef farms in many countries, with GS being more widely used in Europe and MS in North America (Alonso et al. 2013; Wilkinson and Rinne 2018; Dänicke et al. 2020). Dairy farmers in several European countries store more than 90% of their forage production as silage (Alonso et al. 2013). These silages are produced by harvesting and chopping pastures and maize crops, which are subsequently stored under anaerobic conditions by compaction as well as airtight covers, mainly in trench/bunker silos and round bales (Resch et al. 2017).

In Austria, approx. 22% of the dairy farms feed cows a silage-free diet in order to match the haymilch (in German: “heumilch”) standards, which does not allow the feeding with any kind of silage (BMLRT 2021). However, currently, most of the Austrian dairy farms feed their herds with silage year-round or seasonal green fodder plus silage. It has been estimated that Austrian dairy farms present an annual average intake of 3300 kg dry matter (DM)/cow/year of GS and 1200 kg DM/cow/year of MS (FAO, IDF, IFCN 2014). In 2019, 154,769 ha of grassland/pastures (primarily grasses, clovers and lucerne) and 85,684 ha of maize for silage were available for forage production in Austria. In practice, about 75% of the basic fodder is preserved by ensiling, which corresponded to approx. 2.55 million t DM of GS and 1.3 million t DM of MS in 2019 (Resch et al. 2021). Since the economic and dietary relevance of these silages for the cattle industry has been recognized, detailed information on safety concerning natural contaminants (such as mycotoxins) is required (Gallo et al. 2015a).

Despite its crucial role in livestock nutrition, silage quality assessment is often based only on chemical analysis (nutritional composition) without an additional evaluation of the occurrence of pathogenic/toxigenic microorganisms or toxins (Wambacq et al. 2016). Fungi and especially their toxic secondary metabolites—mycotoxins —have been shown to pose a health risk to ruminants, with silages as one of the main sources of exposure (Driehuis et al. 2008a; Ogunade et al. 2018). The fungal toxins produced on-field can persist during the ensiling process, endangering the feed safety (Storm et al. 2014). Even though the ensiling process inactivates most of the microorganisms involved in silage spoilage, some species of filamentous fungi such as *P. roqueforti*, *A. fumigatus*, *M. ruber* and *P. niveus* can tolerate the low pH, high levels of carbon dioxide and low availability of oxygen which occur during storage (Alonso et al. 2013; Wambacq et al. 2016). These moulds can therefore survive in the silos and proliferate when more oxygen is available leading to spoilage, thereby reducing the nutritional value, dry matter content and palatability of the silage. Ultimately, diverse fungi in silage can produce a wide spectrum of secondary metabolites (O'Brien et al. 2006) with different biological activities including immunosuppressive, hepatotoxic, nephrotoxic and neurotoxic effects in animals (Storm et al. 2008; Driehuis et al. 2018). When incorporated into the diets of dairy cows, mouldy silages may impair animal health and productivity (Fink-Gremmels 2008; Santos and Fink-Gremmels 2014). Some evidence suggests that sub-clinical disorders such as impaired rumen function or increased susceptibility to infections might be related to the impact of such complex mixtures of fungal secondary metabolites (Storm et al. 2008; Santos and Fink-Gremmels 2014). Exposure to mouldy feeds seems to induce a poorly characterized sub-clinical disorder described as mouldy silage syndrome (Santos and Fink-Gremmels 2014).

Recent research began to recognize possible synergistic interactions and consequences of long-term exposure to such mycotoxin mixtures and the importance of holistic and innovative approaches based on multi-mycotoxins analyses (Storm et al. 2014; Battilani et al. 2020). So far, research related to this topic has covered the study of fungal populations in silages (Alonso et al. 2013; Rodriguez-Blanco et al. 2020). Additionally, preharvest multi-mycotoxin surveys in maize (Hajnal et al. 2020; Kos et al. 2020) and grasses (Nichea et al. 2015; Penagos-Tabares et al. 2021) as well as postharvest in GS and MS have been carried out (Rasmussen et al. 2010; Shimshoni et al. 2013; Storm et al. 2014; Vandicke et al. 2019; Panasiuk et al. 2019; Reisinger et al. 2019; Rodríguez-Blanco 2019; Dänicke et al. 2020). However, research focused on a wide spectrum of storage-associated mycotoxins in mouldy silages is scarce and the risks of dietary contamination with mouldy spots of silage are not known. Furthermore, several studies suggested that MS represents a higher mycotoxicological risk compared to GS (Panasiuk et al. 2019; Reisinger et al. 2019; Dänicke et al. 2020). Therefore, this study aimed 1) to characterize the most recurrent spoiling fungal organisms (co-) occurring in GS and MS in Austrian dairy farms using the routinary fungal analysis and 2) to assess broad profiles of mycotoxins and other secondary fungal metabolites (> 400) presented in the mouldy portions of silages. The levels and diversity of mycotoxins and metabolites contained in mouldy spots of both silage types were statistically compared. Additionally, possible interrelationships between fungal counts and levels of mycotoxin/metabolites were investigated.

## Materials and methods

### Sampling procedure

**Fig. 1:**
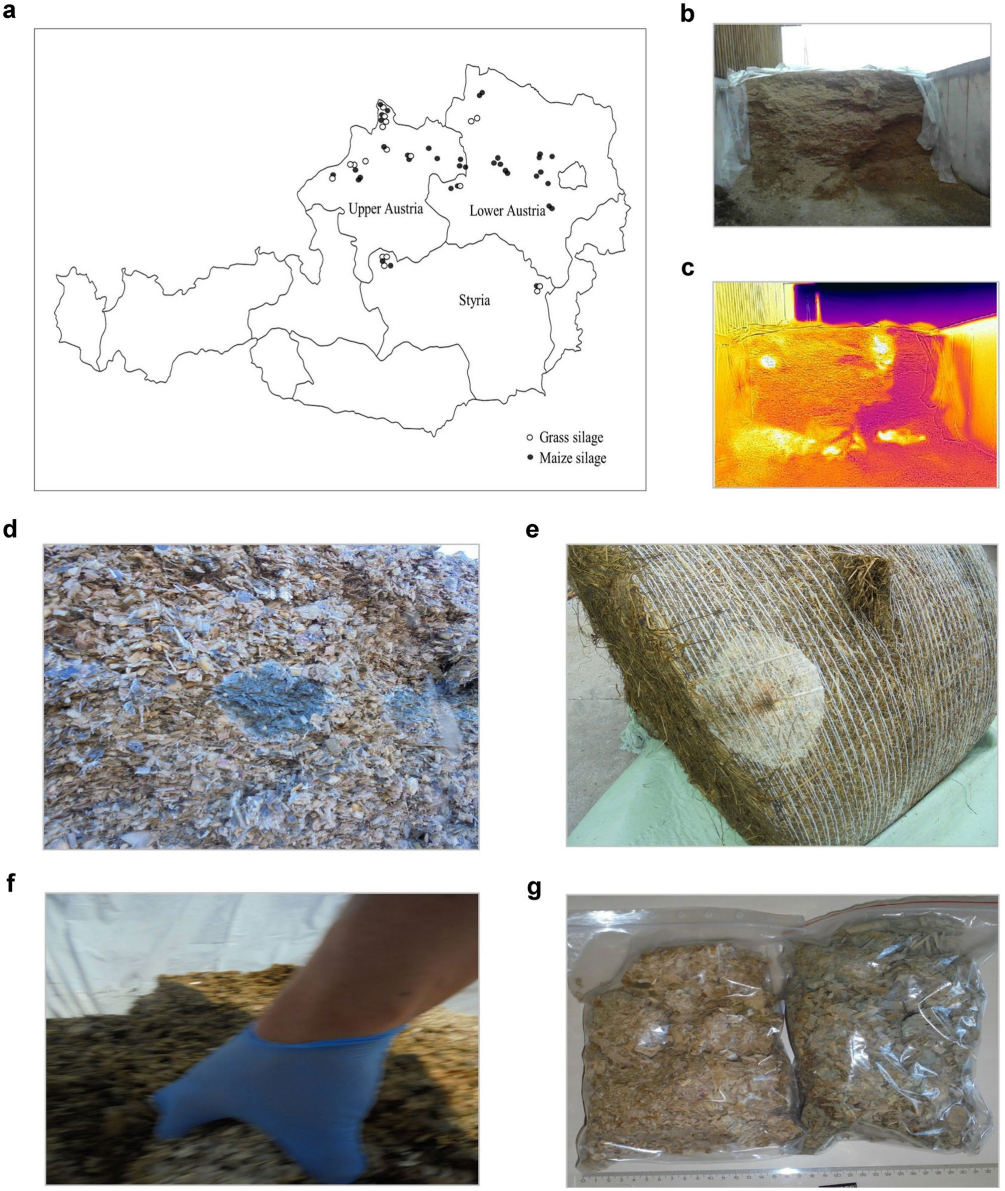
Sampling of mouldy spots of grass and maize silages intended for feeding dairy cows **a** Map of Austria illustrating localization of surveyed samples. **b**, **c** Detection of mouldy spots via infra-red thermography in a ripped round bale of grass silage. **d** Visible mouldy spots of maize silage and **e** grass silage. **f** Sampling manually approx. 500 g of one hotspot with visible fungal growth per silo. Finally, **g** the samples were tightly sealed (the air was squeezed out) and stored at 4 °C in the dark until sample preparation

With the consent of the farmers, samples were collected from a total of 35 dairy farms located in Lower Austria, Upper Austria, and Styria, corresponding to the three Austrian Federal states leading the country’s milk production (Fig. 1a). The samples included in this pilot study were collected between May 2019 and August 2020, totalling 47 samples (19 samples of mouldy spots of GS and 28 of MS) from already opened and “ready to be fed” bunker/trench silos or round bales, which have been ensiled for at least 3 months. We aimed at sampling mouldy spots in silages, and thus, collecting a representative sampling of the complete silo as presented recommended by McElhinney et al. (2016) was not suitable for our goal. Samples from the available silos or bales fitting the aforementioned criteria across the pilot farms were collected and treated as individual samples independently as a means to account for the heterogeneity of the mouldy spots. Sections of silage with evidently dense fungal growth were detected via visual inspection (Fig. 1b) or by using thermal Imaging Camera FLIR ONE and FLIR Tools software (FLIR, Wilsonville, United States) (Fig. 1c). Per silo or bale, a subsample of a spot infested with apparent fungal growth (corroborated by observation of mycelial structures, Fig. 1e-d) was selected for sampling. Such mouldy hot spots were located in the superior and lateral sides of the trench/bunker silos and bales. The sampling consisted of the manual collection of one subsample of approx. 500 g on a wet weight basis of silage from one densely and compactly mould-colonized spot using nitrile gloves, superficially, not deeper than 20 cm (Fig. 1f). Each sample was subsequently stored in plastic bags, which were tightly sealed (the air was squeezed out) (Fig. 1g) and stored at 4 °C in the dark until arriving at the laboratory. Each sample of moulded silage was homogenized using a knife mill (Retsch GmbH, Haan, Germany; Type: GM200) at 10000 rpm for 10 s. Subsequently, 100 g was randomly selected for mycological evaluation and the remaining sample (approximately 400 g) was stored in the dark at -20 °C until further mycotoxin analysis.

### Fungal identification (Plate Counting)

For mycological analysis, 20 g of the sample was mixed with180 ml of 0.1% peptone solution (achieving a 10–1 dilution) and further diluted until 10^–4^. Dilution plating was carried out according to Samson et al. (2019), utilizing selective mycological media, namely, Malt Extract Agar (MEA; Merck, Darmstadt, Germany) supplemented with 100 μg/ml of chloramphenicol (Roth, Karlsruhe, Germany) and Dichloran Rose Bengal Chloramphenicol Agar (DRBC; Roth, Karlsruhe, Germany). These media have been used in studies of mycology of silages (O'Brien et al. 2005; O'Brien et al. 2007; Manfield and Kuldau 2007). For inoculation of the plates, 0.1 mL aliquots representing 10^–2^, 10^–3^ and 10^–4^ dilutions were used, in triplicates. Plates were incubated at 25 °C for 5–7 days in the dark. Additional cultivation at 37 °C for 5 days was used for the isolation of opportunistic fungal pathogens. Each fungal colony isolated from a sample was considered as an individual isolate. Morphological identification of dominant fungal genera/species was performed by evaluation of macro- and microscopic morphological traits according to Samson et al. (2019) and de Hoog et al. (2020).

### Multi-Mycotoxin analysis (LC–ESI–MS/MS)

For mycotoxin analysis, the frozen and previously milled sub-samples (approx. 400 g) were thawed for 12 h and subsequently dried at 65 °C in a ventilated oven for 48 h. Subsequently, the samples were milled through a 0.5-mm sieve using a cutting mill (SM 300, Retsch GmbH, Haan, Germany) at 1,500 rpm during approx.1 min. Five grams (± 0.01 g) of the homogenized samples were added to 50-ml polypropylene conical tubes (Sarstedt, Nümbrecht, Germany) and stored at − 20 °C until analysis. Glacial acetic acid (p.a.) and ammonium acetate (LC–MS grade) were purchased from Sigma-Aldrich (Vienna, Austria), HiPerSolv Chromanorm HPLC gradient grade acetonitrile was obtained from VWR Chemicals (Vienna, Austria), and LC–MS Chromasolv grade methanol was acquired from Honeywell (Seelze, Germany). Water was purified by reverse osmosis utilizing a Purelab Ultra system (ELGA LabWater, Celle, Germany). Standards of > 600 fungal and other secondary metabolites were acquired either via a donation from various research institutions or purchased from several commercial suppliers (Sulyok et al. 2020). Quantitative analysis of all relevant mycotoxins and other secondary metabolites was performed using a validated method based on liquid chromatography-electrospray ionization tandem mass spectrometry (LC–ESI–MS/MS) described by Sulyok et al. (2020). Briefly, 5 g of milled sample was deposited into a 250 ml Erlenmeyer flask along with 20 ml of extraction solvent. It was agitated for 90 min using a GFL 3017 rotary shaker (GFL, Burgwedel, Germany). Subsequently, the mixture was centrifuged for 2 min at 2,012 × g on a GS-6 centrifuge (Beckman Coulter Inc., Brea, CA, USA). The extract was transferred into glass vials and diluted 1:1 with dilution solvent. The injection volume of both raw extracts of the samples and the mycotoxin standard solutions was 5 µl. Identification and quantification of each mycotoxin were performed in the scheduled multiple reaction monitoring (sMRM) mode both in positive and negative polarity in two separate chromatographic runs using a QTrap 5500 LC–MS/MS system (Applied Biosystems, Foster City, CA, USA) equipped with a TurboV electrospray ionization (ESI) source was coupled to a 1290 series UHPLC system (Agilent Technologies, Waldbronn, Germany). Chromatographic separation was accomplished by binary gradient elution. Quantification was based on external calibration using a serial dilution of a multi-analyte stock solution. Results were corrected for apparent recoveries determined during method validation according to Steiner et al. (2020). The accuracy of the method is verified by participation in a proficiency testing scheme with > 95% of the > 1600 results submitted so far exhibiting z-scores between -2 and 2. In particular, 15 out of 16 parameters submitted for a sample of whole-plant MS were in the satisfactory range with the exception being zearalenone (z = -2.04). The method used here has been employed to study multi-mycotoxin occurrence in diverse complex matrices of feedstuffs such as silage, pastures, concentrate feed and total mix rations (Shimshoni et al. 2013; Nichea et al. 2015; Kemboi et al. 2020; Penagos-Tabares et al. 2021; Awapak et al. 2021).

### Statistical analysis

Occurrences and the descriptive statistics, i.e. minimum–maximum concentrations, median and mean values of the concentration of metabolites were calculated considering only the positive values (x ≥ limit of detection (LOD)). Concentrations of metabolites were presented on a dry matter basis in μg/kg. Values under the limit of quantification (LOQ) were computed as LOQ/2. To assess the significance of the differences between fungal counts and levels of mycotoxins and additional metabolites in mouldy GS and MS, a Mann–Whitney rank-sum test was performed, and statistical differences were considered significant at *p*-value < 0.05. A two-tailed Spearman’s correlation test was conducted to explore possible relationships between fungal counts and levels of metabolites as well as relationships among metabolites within each kind of silage. For this, only data of metabolites with occurrence over 30% were considered. Spearman’s correlation coefficients were considered significant at *p*-value < 0.05, and the interpretation was performed according to Schober et al. (2018). Accordingly, the correlation coefficients were considered significant at level *p*-value < 0.01 and the magnitude of the observed correlation was interpreted as “very strong” (0.90 up to 1.00), “strong” (0.70 up to 0.89) and “moderate” (0.40 up to 0.69) according to Schober et al. (2018). Linear regressions between fungal metabolites were performed to corroborate the promising relationships. The mentioned statistical analyses and graphs were performed using GraphPad Prism version 9.1 (GraphPad Software, San Diego, California, USA) and Microsoft^®^ Excel^®^. Additionally, an effect of the occurrence of dominant mould species *P. roqueforti* on the concentration of *Penicillium* spp. metabolites was determined. For this purpose, the counts were classified into four groups: no (zero counts, *n* = 13), low (1 × 10^4^ CFU/g – 5 × 10^5^ CFU/g, *n* = 19), medium (1 × 10^6^ CFU/g – 5 × 10^6^ CFU/g, *n* = 9), and high (1 × 10^8^ CFU/g, *n* = 9). Data were subsequently tested using a mixed model consisting of the fixed effect of the *P. roqueforti* group and the random effect of the kind of silage. The mixed model was analysed using PROC MIXED of SAS (version 9.4; SAS Institute Inc., Cary, NC, USA). Pairwise comparisons of the resulting least-squares means were done using the PDIFF option, and significance was declared at *p*-value < 0.05.

## Results

### Occurrence and counts of fungal organisms

**Fig. 2:**
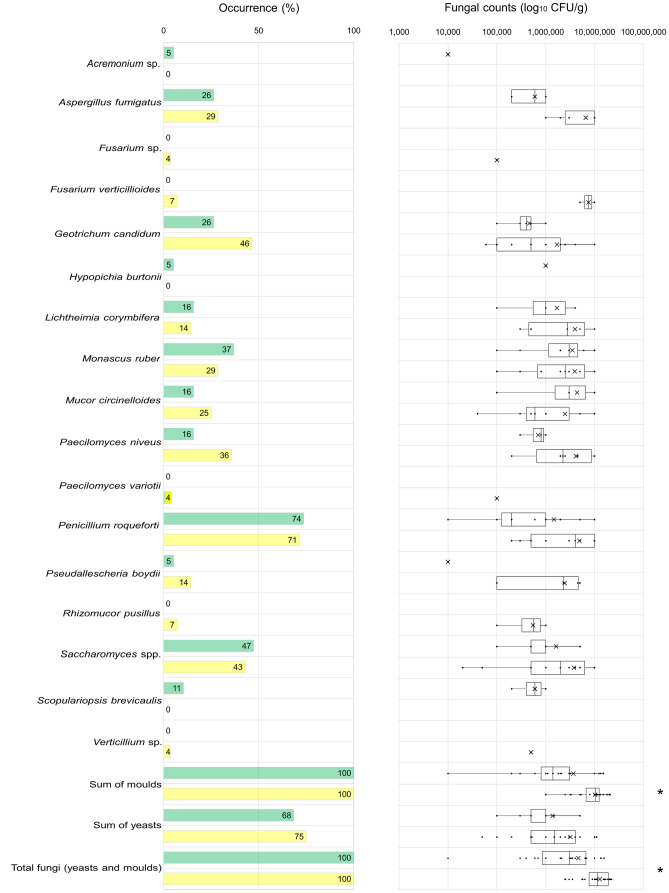
Occurrences and counts (CFU/g) of fungal species isolated from mouldy grass (green) and maize silages (yellow)* Significantly different (*p*-value < 0.05)

Seventeen distinct fungal organisms were detected in mouldy silages, consisting of 3 yeasts and 14 moulds identified at species or genus level (Fig. 2). Respectively, 12 different fungi in GS and 14 in MS were isolated. All samples were positive for moulds, whereas for yeasts only 68% and 75% of GS and MS were positive, respectively. The mould *P. roqueforti* was the most frequently isolated fungi in both types of mouldy silage, occurring specifically in 74% of GS and 71% of MS samples. For GS, the most common fungi were *Saccharomyces* spp. (47%), *M. ruber* (37%), *A. fumigatus* (26%), *G. candidum* (26%), *M. circinelloides* (16%), *Lichtheimia* (formerly *Absidia*) *corymbifera* (16%), *P. niveus* (formerly *Byssochlamys nivea*) (16%) and with lower incidence *Scopulariopsis brevicaulis* (11%) and *Hypopichia burtonii* (5%) as well as *Acremonium* sp. (5%). After *P. roqueforti,* MS samples were mostly contaminated with *G. candidum* (46%), *Saccharomyces* spp. (43%), *P. niveus* (36%), *A. fumigatus* (29%), *M. ruber* (29%), *M. circinelloides* (25%), *L. corymbifera* (14%) and *Pseudallescheria boydii* (14%). With occurrences under 10%, *Rhizomucor pusillus*, *F. verticillioides, Fusarium* spp., *Paecilomyces variotii* and *Verticillium* sp. were detected exclusively in MS. As shown in Supplementary Figure S1, in mouldy GS, *P. roqueforti* frequently co-occurred with *Saccharomyces* spp. (32%), *G. candidum* (21%), *M. ruber* (16%) and *A. fumigatus* (16%), while *Saccharomyces* spp. co-occurred with *A. fumigatus* (26%) and *M. ruber* (26%) as well as *M. ruber* with *A. fumigatus* (16%) and *L. corymbifera* (16%). In mouldy MS, *P. roqueforti* frequently co-occurred with *P. niveus* (32%), *G. candidum* (29%), *M. ruber* (29%) and *M. circinelloides* (18%), along with *M. ruber* and *P. niveus* (18%) (Supplementary Figure S1).

Mouldy spots of MS presented significantly superior total fungal counts, i.e. the sum of moulds and yeasts (*p*-value < 0.001) and total mould counts (*p*-value < 0.001) but not total yeast counts compared with the GS (Fig. 2). Total fungal count ranged from 1 × 10^4^ CFU/g to 1.5 × 10^7^ CFU/g in samples of GS and from 2.5 × 10^6^ CFU/g to 2.2 × 10^7^ CFU/g in MS (Fig. 2, Supplementary Table S1). No statistical differences between GS and MS were observed for the counts of other identified fungal organisms. The highest counts in mouldy GS were *M. circinelloides*, followed by *M. ruber*, *L. corymbifera*, *P. roqueforti* and *H. burtonii*, which presented average counts of over 1 × 10^6^ CFU/g. Compared to mouldy GS, the analysed MS samples displayed superior average counts of *P. boydii*, *P. roqueforti*, *P. variotii*, *M. circinelloides*, *M. ruber*, *H. burtonii* and *G. candidum, F. verticillioides* and another *Fusarium* sp.

### Occurrence and concentrations of mycotoxins and other secondary metabolites

#### General overview

**Fig. 3:**
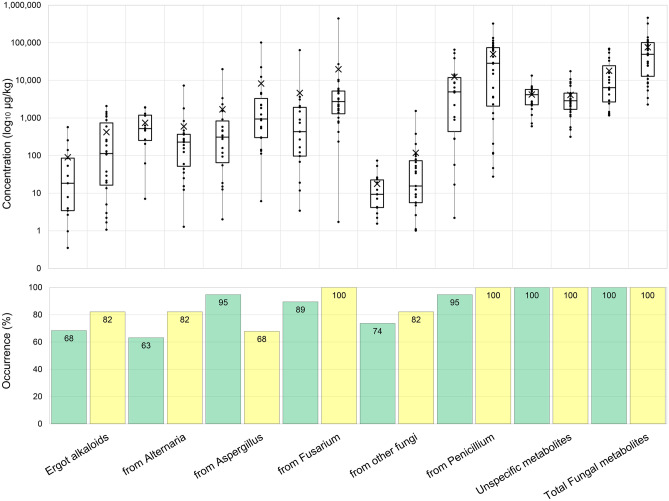
Occurrences and concentration of grouped mycotoxins, other fungal and unspecific metabolites detected in mouldy spots of grass (green) and maize silages (yellow)* Significantly different (*p*-value < 0.05)

A total of 106 and 83 secondary metabolites were detected across all MS and GS samples, respectively (Supplementary Table S1). To simplify the results’ presentation along with their interpretation, the detected metabolites were classified by major producers based on previous reports with some modifications (Szulc et al. 2019; Hajnal et al. 2020; Penagos-Tabares et al. 2021) in the following categories: *Alternaria* spp. (5), *Aspergillus* spp. (23), *Fusarium* spp. (32), *Penicillium* spp. (16), other fungi (8), unspecific (19) and ergot alkaloids (EAs) (3). Figure 3 illustrates the occurrences and concentrations (mean, median, maximum and minimum) of the mentioned groups. Among the identified producers, metabolites mainly produced by *Penicillium* spp. were the most frequently detected and were found in all the samples of mouldy MS and 95% of GS. The highly diverse fusarial metabolites were positive in 100 and 89% of mouldy MS and GS, respectively. Diverse metabolites from *Aspergillus* spp. were also evident (Supplementary Table S1) but were detected in a lower frequency across the evaluated samples (82% in MS and 63% in GS, Fig. 3). Lower numbers of EAs metabolites, as well as metabolites derived from genus *Alternaria*, and other fungi (Supplementary Table S1) were detected in over 60% of the evaluated samples (Fig. 3). When comparing the two silages, MS samples presented significatively higher levels of total EAs (*p*-value = 0.045) as well as of total metabolites derived from *Fusarium* spp. (*p*-value < 0.001), *Penicillium* spp. (*p*-value = 0.017) and fungi (*p*-value < 0.001). All samples contained considerable amounts of unspecific metabolites, ranging from 602 µg/kg to13,400 µg/kg in GS and from 316 µg/kg to 17,500 µg/kg in MS (Fig. 3).

#### Selected mycotoxins and fungal metabolites

The occurrence, concentrations (mean, median and range) as well as the differences of selected mycotoxins levels between both kinds of silages are presented in Table 1. Other less known and lower recurrent mycotoxins and metabolites are given in Supplementary Table S3. Regarding mycotoxins contemplated in European legislation, GS samples presented relatively low frequencies (16% and 21%) of deoxynivalenol (DON) and zearalenone (ZEN) in comparison with the MS samples that were over 60% positive for both mycotoxins. Despite the low occurrence in GS, the maximum concentration of ZEN (668 µg/kg) exceeded the EU guidance level of 500 µg/kg (for ZEN in complementary and complete feedingstuffs for dairy cattle) (EC 2006), whereas ZEN ranged only from 2.08 µg/kg to 53.9 µg/kg in MS. All samples were negative for aflatoxin B1, ochratoxin A and T-2 toxin.

**Table 1:** Occurrences and levels of selected mycotoxins and metabolites detected in spots of mouldy grass and maize silages

		**Grass silage (*n* = 19)**								**Maize silage (*n* = 28)**							
**Group**	**Metabolites**	**Positive Samples (%)**^**a**^	**Concentration**^**b**^							**Positive Samples (%)**^**a**^	**Concentration**^**b**^						
			**Average ± SD**			**Median**	**Range**				**Average ± SD**			**Median**	**Range**		
**Ergot alkaloids**	**Agroclavine**	11	2.47	±	0.3	2.47	2.25	-	2.68	32	8.06	±	7.35	6.43	1.44	-	23.1
	**Chanoclavine**	58	36.5	±	73.6	5.78	0.16	-	225	54	160	±	445	18.7	0.17		1740
	Festuclavine*	63	63.7	±	123	14.9	0.35	-	435	82	313	±	444	86.9	1.07	-	1360
***Alternaria* spp.**	**Alternariol**	16	10.2	±	12.2	4.35	2.09	-	24.2	29	2.34	±	3.36	1.14	0.3	-	10.4
	**Alternariolmethylether**	26	4.13	±	2.3	3.5	1.6	-	7.32	29	1.78	±	1.62	1.31	0.13	-	4.71
	**Altersetin**	32	176	±	315	58.1	5.13	-	818	36	13.6	±	9.85	12.6	1.11	-	31.6
	**Tenuazonic acid**	53	781	±	552	569	195	-	1920	61	785	±	1720	275	57.2	-	7270
	**Averufin**	21	2.75	±	2.64	2.08	0.34	-	6.51	7	2.01	±	0.78	2.01	1.46	-	2.56
***Aspergillus* spp.**	**Bis(methylthio)gliotoxin**	11	133	±	184	133	2.19	-	263	32	152	±	242	63.8	6.53	-	756
	**Fumigaclavine**	26	276	±	557	5.34	1.56	-	1270	32	563	±	729	212	1.08	-	2040
	**Fumigaclavine C**	37	1800	±	4000	81.3	11.3	-	10,780	36	3950	±	7430	857	5.61	-	23,290
	**Gliotoxin**	5						79.3		14	47	±	46.6	46.8	5.09	-	89.2
	**Helvolic acid**	5						131		18	406	±	703	76.4	53.4	-	1660
	**Kojic acid**	21	63.2	±	44.5	43.5	36.2	-	129	43	97.7	±	103	54.3	16.1	-	353
	Sterigmatocystin*	37	6.89	±	9.79	1.3	0.09	-	26.6	7	2.49	±	3.2	2.49	0.23	-	4.75
***Fusarium* spp.**	15-Hydroxyculmorin*	5						16.3		46	143	±	192	76.5	33.7	-	742
	**alpha-Zearalenol**									11	62.1	±	56.3	61.4	6.08	-	119
	Apicidin*	5						7.92		71	23.8	±	25.4	17.2	3.81	-	111
	Aurofusarin*	32	35.5	±	23.2	41.2	4.07	-	59.9	75	61.8	±	50.5	41	3.92	-	171
	Beauvericin*	47	19.7	±	40.4	1.83	0.2	-	125	86	30.1	±	36.7	17.7	3.93	-	153
	**Bikaverin**									46	9.7	±	5.68	7.04	3.53	-	22.7
	**Chrysogine**	53	34.4	±	31.8	23.1	4.61	-	102	29	6.64	±	5.07	4.44	2.35	-	15.8
	**Culmorin**	42	83.8	±	65.1	62.7	5.77	-	179	79	302	±	366	199	20.7	-	1360
	**Deoxynivalenol**	16	19.6	±	10.2	20	9.24	-	29.6	79	291	±	285	224	30	-	1220
	**Enniatin A**	37	1.36	±	1.75	0.81	0.02	-	4.9	43	0.85	±	0.81	0.67	0.01	-	2.17
	**Enniatin A1**	58	3.89	±	6.01	2.2	0.17	-	20.3	75	10.9	±	14.3	4.27	0.2	-	51.4
	**Enniatin B**	84	11.1	±	13.3	6.37	0.27	-	44.5	86	8.26	±	10.5	4.94	0.11	-	44.7
	**Enniatin B1**	68	12.6	±	21.1	7.19	0.64	-	80.7	68	19.4	±	26.8	7.16	0.05	-	95.3
	**Enniatin B2**	26	0.7	±	0.79	0.44	0.14	-	2.08	32	0.46	±	0.32	0.42	0.11	-	1.06
	**Epiequisetin**	37	7.15	±	8.45	2.65	1.01	-	22.3	46	6.42	±	6.95	3.45	0.3	-	23.5
	**Equisetin**	47	39.4	±	67.1	8.55	0.65	-	181	46	9.16	±	11.2	4.12	1.23	-	41.9
	**Fumonisin B1**									75	88.4	±	79.0	58.8	14	-	356
	**Fumonisin B2**									50	28.7	±	22.4	25.6	10.1	-	97.8
	**HT-2 toxin**									21	16.8	±	9.65	14.6	4.81	-	31
	**Moniliformin**	5						8.47		29	5.76	±	5.06	4.46	1.56	-	17
	**Nivalenol**	5						*36*		89	281	±	219	191.1	38.9	-	852
	**Siccanol**	47	8130	±	20,680	1400	200	-	63,200	82	3200	±	5380	1580	154	-	26,100
	**Zearalenone**	21	178	±	327	20.2	3.43	-	668	61	15	±	14.4	10.6	2.08	-	53.9
***Penicillium spp.***	**Andrastin A**	84	1030	±	1850	90.8	4.02	-	5840	86	3860	±	4160	2170	19.6	-	13,100
	**Andrastin B**	74	508	±	718	140	6.96	-	2270	79	3670	±	4300	1900	5.81	-	14,100
	**Andrastin C**	84	9580	±	14,800	723	71.3	-	36,720	79	45,200	±	58,100	32,800	21.5	-	252,100
	**Marcfortine A**	63	201	±	531	16.7	4.11	-	1880	68	2030	±	3060	777	1.01	-	12,900
	**Mycophenolic acid**	79	2530	±	2740	1960	18.1	-	7450	82	5570	±	9130	2000	2.59	-	30,900
	**Mycophenolic acid IV**	63	108	±	155	57.1	1.57	-	570	68	199	±	307	50.5	0.41	-	1050
	**Questiomycin A**	11	27.4	±	29.6	27.4	6.46	-	48.3	64	27.3	±	33.1	15.2	4.24	-	111
	**Roquefortine C**	79	2270	±	2940	1150	64.5	-	10,900	86	6360	±	6080	6530	6.36	-	20,000
	**Roquefortine D**	58	756	±	1340	160	32.7	-	4400	50	6220	±	9690	1970	129	-	31,200

*Significantly different (*p*-value < 0.05)

^a^Samples with values > limit of detection (LOD)

^b^Excluding data < LOD. In case values > LOD and < limit of quantification (LOQ), LOQ/2 was used for calculation

The fusarial mycoestrogen, alpha-zearalenol (α-ZEL) (11% occurrence) along with HT-2 toxin (21%), types B of fumonisins (FB) (1,2,3, and 4) (75%, 50%, 11% and 11%, respectively), nivalenol (NIV) (89%), fusaric acid (FA) (18%), butanolide (14%) and monoacetoxyscirpenol (MAS) (4%) were detected only in MS (Table 1). The most recurrent *Fusarium*-related mycotoxin in GS belonged to the enniatin (ENN) group: ENN B (84%), ENN B_1_ (68%) and ENN A1 (58%). In MS, DON, NIV and FB_1_, ENN A and B, beauvericin (BEA), siccanol, culmorin, aurofusarin and apicidin occurred in over 70% of the samples. The metabolites related to *Fusarium* spp. with the highest average concentrations were siccanol (8130 µg/kg) and fusaric acid (83300 µg/kg). In comparison with GS, MS samples showed significantly superior levels of DON (*p*-value < 0.001), NIV (*p*-value < 0.001), FB_1_ (*p*-value < 0.001), FB2 (*p*-value < 0.001), ENN A_1_ (*p*-value = 0.041), BEA (*p*-value < 0.001), aurofusarin (*p*-value = 0.004), bikaverin (*p*-value < 0.001), culmorin (*p*-value < 0.001), and apicidin (*p*-value < 0.001). Interestingly, the concentrations of siccanol (*p*-value = 0.015), ZEN (*p*-value = 0.0162) and chrysogine (*p*-value = 0.016) were significatively higher in GS samples.

Regarding *Penicillium*-derived metabolites, andrastins (AND) A, B, and C, marcfortine A, mycophenolic acid (MPA), MPA IV as well as roquefortines (ROQ) C and D were found in both silages in frequencies ≥ 50% (Table 1). Citrinin was detected only in one GS sample (99.7 µg/kg). The *Penicillium* mycotoxins with highest average concentrations in GS samples were AND C (9580 µg/kg), MPA (2530 µg/kg), ROQ C (2270 µg/kg) and AND A (1030 µg/kg). For MS samples, the metabolites with highest average concentrations were AND C (45,200 µg/kg), ROQ C (6360 µg/kg), ROQ D (6220 µg/kg), MPA (5570 µg/kg), AND A (3860 µg/kg), AND B (3670 µg/kg) and MAC A (2030 µg/kg). The samples of MS presented significantly higher concentrations of AND A (*p*-value = 0.003), questiomycin A (*p*-value < 0.001) and chevalone C (*p*-value < 0.001) compared to those in GS samples. The metabolite pestalotin was detected only in mouldy MS (Table 1, Supplementary Table S3).

Three clavine alkaloids were found both silages: festuclavine (FES) (MS:82%, GS:63%), chanoclavine (MS:54%, GS:58%) and agroclavine (MS:32%, GS:11%) (Supplementary Table S2). The concentrations of these EAs were generally higher in MS compared to GS, but only FES (most produced EA in both groups of silages) reached significance (*p*-value = 0.026) (Supplementary Table S2). Tenuazonic acid (TeA) was the most frequent mycotoxin produced by *Alternaria* spp. detected in both GS and MS (53% and 61%, respectively) and with a lesser frequency alternariol (AHO) and alternariol-methyl-ether (AME) (< 40% and the concentrations under 1000 µg/kg) (Table 1). Both silage groups did not differ in the concentration of *Alternaria*-derived compounds.

Regarding *Aspergillus*-derived metabolites, the mycotoxins sterigmatocystin (STC), bis(methylthio)gliotoxin, gliotoxin, fumiquinazolines (FQ) A and D, fumigaclavine (FM) and fumigaclavine C (FMC) were detected. Their occurrences were under 40% for both GS and MS. FQA and FMC were the *Aspergillus-*derived mycotoxins with the highest average concentrations (over 3800 µg/kg) in MS. Despite having a higher average, sphingofungin B (7250 µg/kg) was found at a lower frequency (11%) (Supplementary Table S2). Likewise, GS samples also presented a predominant production of FMC and FQA, corresponding to average concentrations of 1800 µg/kg and 433 µg/kg. Interestingly, GS showed significantly higher contamination levels of SCT than MS (*p*-value = 0.0113) (Table 1). Other metabolites produced by other fungi and by organisms from other kingdoms (such as Bacteria and Plantae) are included in the Supplementary Table S1. Metabolites designated mycotoxins but also produced by plants, such as emodin (GS:95%, MS: 89%) and 3-Nitropropionic acid (GS:26%, MS: 54%) were also detected (Table 1). Differences between the mycotoxin content in mouldy MS and GS during the years 2019 and 2020 were analysed via Mann–Whitney Test. The metabolites with significant differences and the respective concentrations (average and median) are listed in the supplementary Table S3.

#### Co-occurrence analysis of mycotoxins and other fungal metabolites

**Fig. 4:**
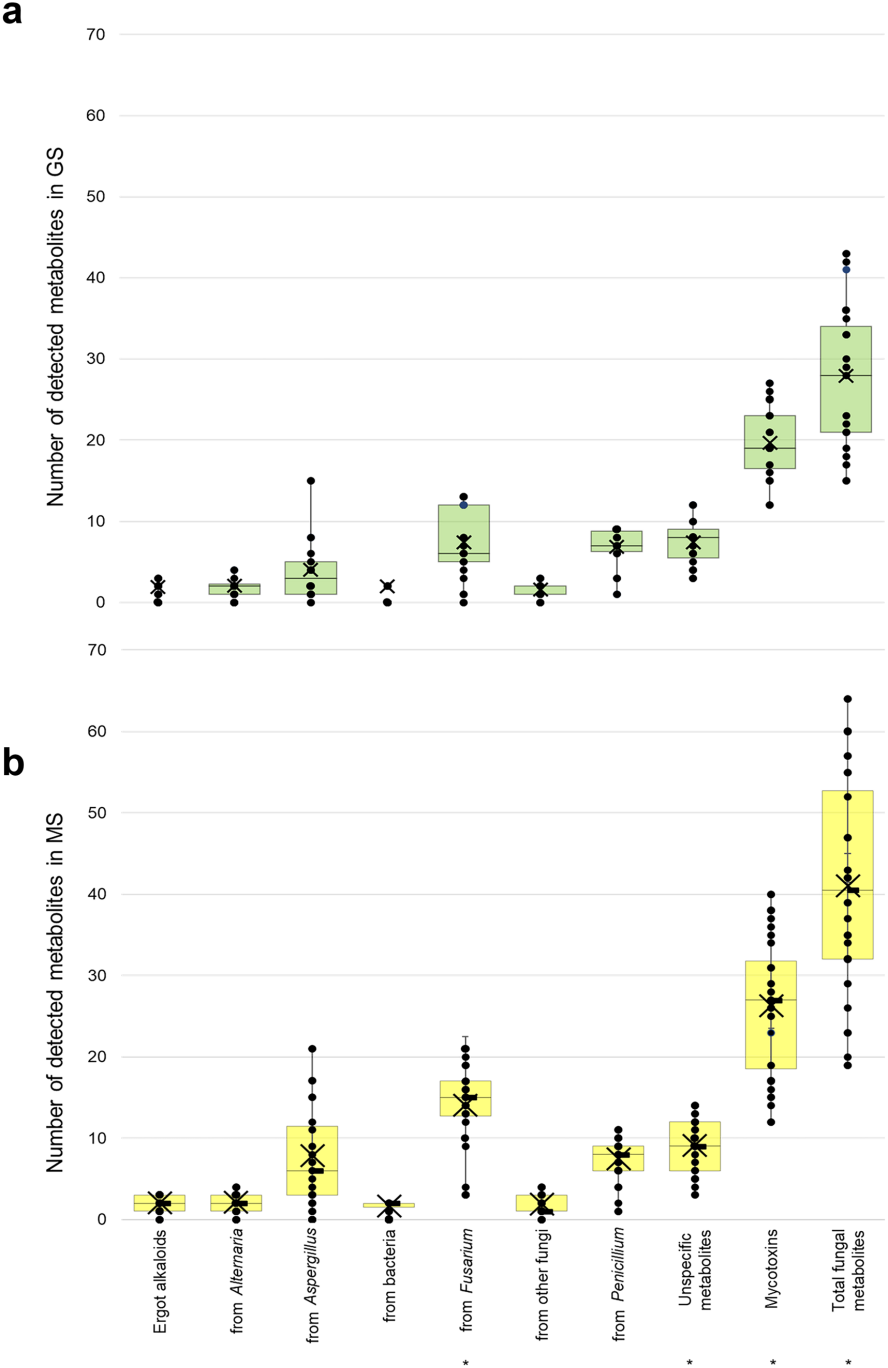
Co-occurrence of grouped mycotoxins, other fungal and unspecific metabolites detected in mouldy spots of grass (green) and maize silages (yellow)* Significantly different (*p*-value < 0.05)

**Fig. 5:**
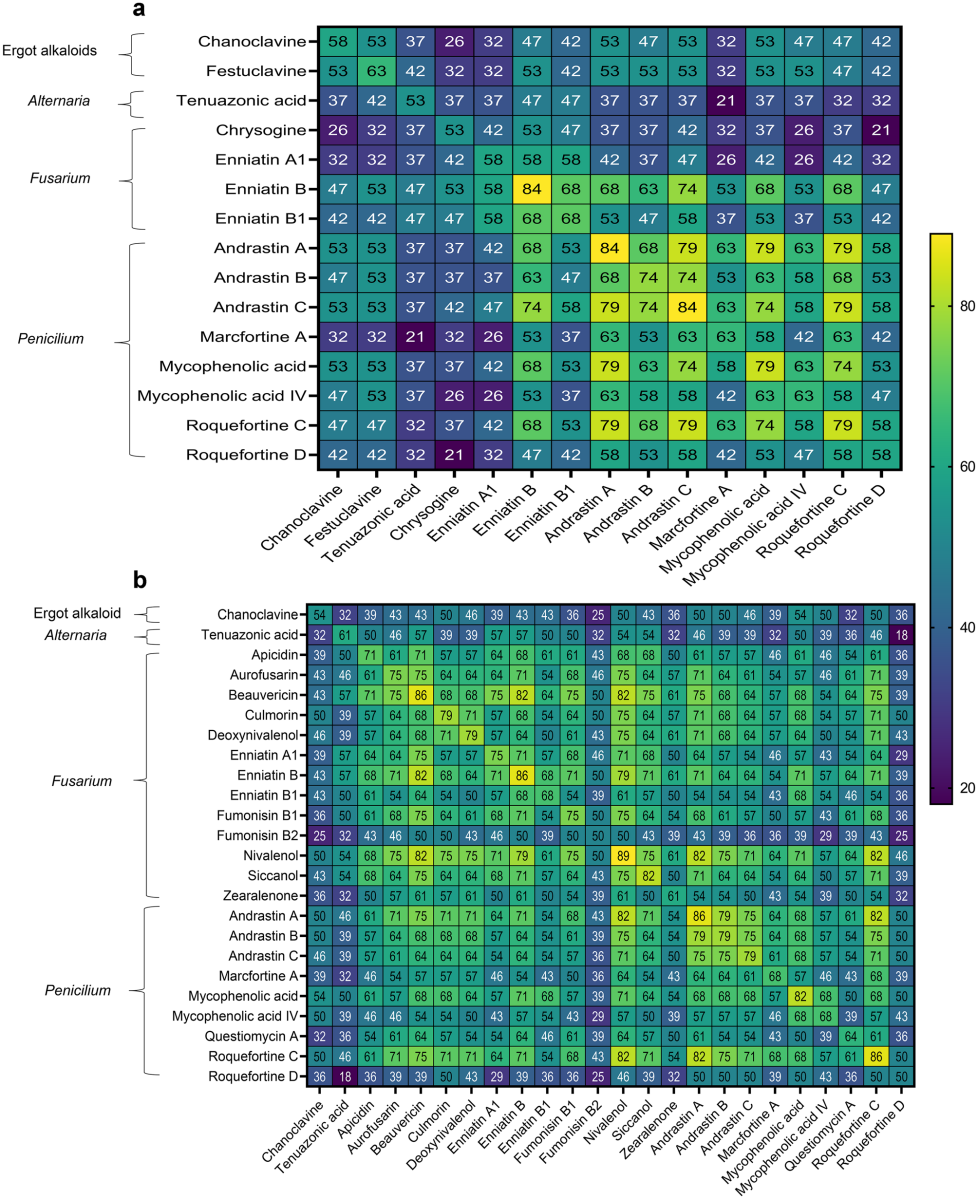
Heatmap of the most frequent mycotoxins combinations (%) detected in mouldy spots of **a** grass and **b** maize silage. Mycotoxins include in this analysis occurred in ≥ 50% of the samples

All samples were co-contaminated with several mycotoxins and other fungal metabolites. Figure 4 shows the average, median and range of co-contamination (i.e. the number of metabolites detected per sample) of different groups of metabolites per silage type. GS had an average of 20 mycotoxins, with samples ranging from 12 to 27, whereas MS presented a mean of 26, varying from 19 to 64 mycotoxins. The number of *Fusarium* spp. metabolites (*p*-value < 0.001), total fungal metabolites (*p*-value < 0.001) and total mycotoxins (*p*-value < 0.003) was higher in MS than GS. Figure 5 illustrates the most common combinations of mycotoxins detected in GS and MS. Accordingly, the co-occurrence of several combinations of metabolites derived mostly from *Fusarium* spp. and *Penicillium* spp. in both mouldy silages was evident. Particularly in GS, over 50% of the samples presented a combination of ENN B and *Penicillium-*derived toxins AND A, AND B, AND C, ROQ C, MPA and MPA IV. MS also showed co-occurrence of ENNs, NIV, DON, FB1, ZEN ≥ 50%, and many of the previously mentioned toxins produced by *Penicillium* spp.

#### Relationship between fungal counts and concentrations of groups of metabolites

Spearman’s correlations between total counts of fungi, moulds, and *P. roqueforti* and the groups of metabolites were mainly weak in GS. However, in MS, a positive moderate correlation (ρ = 0.68, *p*-value < 0.001) between the counts of *P. roqueforti* and the group of *Penicillium*-derived metabolites was found (*p-*value < 0.05). According to the mixed model analysis, as shown in Supplementary Figure S2a, a significant increase in the concentration of *Penicillium*-derived metabolites (60,000–65,000 µg/kg) was found with the groups with medium (× 10^6^ CFU/g) and high (× 10^8^ CFU/g) counts of *P. roqueforti* compared to the groups with non-detectable counts (0 CFU/g) and the low count group (10^4^ – 10^5^ CFU/g) of *P. roqueforti* (*p*-value < 0.05).

#### Relationship between concentrations and groups of mycotoxins and metabolites

In GS, a strong positive correlation (ρ = 0.81, *p*-value < 0.001) between *Penicillium* spp. metabolites and total fungal metabolites was evident (Supplementary Figure S2b and c). Specifically, the total of *Penicillium*-derived metabolites was strongly correlated with AND A (ρ = 0.81, *p*-value < 0.001), AND B (ρ = 0.82, *p*-value < 0.001), MPA (ρ = 0.72, *p*-value < 0.001), MPA IV (ρ = 0.74, *p*-value < 0.001) and ROQ C (ρ = 0.81, *p*-value < 0.001). However, only AND A, B and C in addition to ROQ C and D showed significance in the regression analysis. A strong relationship (ρ = 0.80, *p*-value < 0.001) between total *Penicillium*-produced and total fungal metabolites) was detected for both MS and GS (Supplementary Figures S2b, c and d). Additionally, metabolites associated with *Aspergillus* spp. presented a moderate relationship (ρ = 0.73, *p*-value < 0.001) with the unspecific metabolites (Supplementary Figure S2e). The mycotoxins DON was strongly correlated with ZEN (ρ = 0.80, *p*-value < 0.001) in MS (Supplementary Figure S2f). The correlation between FES and some of the *Penicillium* spp. toxins and metabolites (AND A, ROQ C and ROQ D) in samples of mouldy GS was confirmed by regression analyses (Supplementary Figure S2g).

## Discussion

Mould contamination and associated mycotoxin production in silages are commonly occurring concerns in dairy farming and animal nutrition since mould growth deteriorates both nutritional and organoleptic properties of silage. Our results reveal the diversity of organisms co-occurring in individual samples of mouldy silages from Austria dairy farms as well as the presence of complex metabolites mixtures, which contain dozens of compounds with toxic or potentially toxic activity. Both toxigenic moulds, e.g. *A. fumigatus*, *P. niveus*, *M. ruber*, *M. circinelloides*, *F. verticilliodes*, and *Acremonium* sp. as well as silage-spoiling non-toxigenic fungi such as yeasts (*Saccharomyces* spp., *G. candidum* and *H. burtonii*) were detected in mouldy spots, which is in accordance with previous reports (Hollmann et al. 2008; Robledo et al. 2016; Wambacq et al. 2016; Rodríguez-Blanco et al. 2019). Silages comprise interesting microbial ecosystems, which can have diverse profiles of secondary metabolites (Alonso et al. 2013). Pre-harvest infestations of *Fusarium* spp., *Alternaria* spp., *Aspergillus* spp. as well as other endophytic symbionts in pastures or cereals, such as *Claviceps* spp. and *Neotyphodium* spp. can generate contamination and accumulation of the so-called field mycotoxins (Driehuis 2013; Driehuis et al. 2018). Our previous work indicated that the natural contamination of pastures with toxins derived mostly from *Fusarium* spp., but also *Alternaria* spp. and *Aspergillus* spp. in addition to EAs (Penagos-Tabares et al. 2021). During the harvesting and chopping processes, additional fungal contamination from the environment (air, soil, and dust) can take place. This newly established microbiota as well as existing field mycotoxins are ensiled together with the chopped raw plant materials (Mansfield and Kuldau 2007). Aerobic conditions and suboptimal silage management promote to the formation of fungi and during the ensiling process or the feeding out. Spots of dense fungal growth (mycelia) can be routinary found in silos intended for livestock feeding worldwide. Such mouldy spots are heterogeneous and not always visible, representing potential sources of mycotoxins and bioactive fungal metabolites that are associated with unspecific syndromes in dairy cattle (Santos and Fink-Gremmels 2014). This exploratory approach was planned to cover a general picture of the dominant cultivable fungi and an extremely wide toxin diversity associated with those visible or thermo-detectable common epicentres of postharvest deterioration. This study indicates that *Penicillium*-derived metabolites presented the highest mean concentrations within mouldy spots of silages, coinciding with the high frequency of *P. roqueforti*, the dominant species in mouldy spots. This supports the previous indication for the main role of *P. roqueforti* in the spoilage and toxin contamination of silages of different countries as reviewed previously (Alonso et al. 2013).

Moreover, we demonstrated that mouldy spots of MS presented a significantly higher diversity and concentration of *Penicillium-*derived toxins than those of GS. These fungal toxins are recognized as the most relevant post-harvest toxins in conserved forages (Pahlow et al. 2003). Various *Penicillium*-derived compounds have been previously detected in silages, such as MPA, ROQs, AND A, agroclavine, marcfortine A (MAC A) and FES (Gallo et al. 2015b; O'Brien et al. 2006, 2008; Storm et al. 2014). The most studied *Penicillium-*derived compounds in ensiled products are MPA and ROQs (Gallo et al. 2015a). These compounds were shown to be more concentrated on the surface layer than in the core of silage (Dreihuis et al. 2008b), and high concentrations of MPA in GS were found in visible aerobic instability and mouldy spots (Santos and Fink-Gremmels 2014), likely due to the proliferation of aerobic fungi *Penicillium* spp*.* In terms of toxicity, ROQ C has been shown to cause neurotoxic effects. The clinical manifestations observed in a herd of cows after the ingestion of grain containing ROQ C (approx. 25,300 µg per kg DM) involved extensive paralysis that did not respond to treatment with calcium. The neurological signs disappeared as soon as the cows were no longer fed with mouldy grain (Häggblom 1990). In our study, the most common *Penicillium* spp. mycotoxins and metabolites co-occurring in both mouldy silages were AND C, followed by ROQ C, ROQ D, MPA, AND B, AND C and MAC A. Notably, the more diverse *Penicillium-*derived metabolites in MS compared to GS cannot be explained by the counts of *P. roqueforti.* Furthermore, the counts of *P. roqueforti* were positively correlated with the concentration of *Penicillium* spp. metabolites in MS but not in GS. The incidence of feed contamination with *Penicillium* spp. reported in the literature is variable (Auerbach et al. 1998; Gallo et al. 2015a; Mansfield et al. 2008; O'Brien et al. 2006), and there is not enough data to link *Penicillium* spp. and their produced metabolites. However, different profiles of metabolites could result from the same species depending on the high variability of strains and sometimes lack of adequate growing conditions. For example, previous studies showed that different strains of *P. roqueforti* isolated from mouldy GS and cultured in vitro presented remarkable differences in the profiles of mycotoxins produced (O'Brien et al. 2006, 2008). The agricultural and economic relevance of *Penicillium* spp. mycotoxins is considered underestimated since they are believed to be rapidly metabolized by gut microbiota and hepatic enzymes (Fuchs et al. 2008; Oh et al. 2013, 2015), but the detoxification process of mycotoxins can be disrupted by their antimicrobial and hepatotoxic properties (Noto et al. 1969; Kopp-Holtwiesche and Rehm 1990; Bentley 2000; Oh et al. 2015).

In the current study, many toxins were detected in the mouldy spots of silages, including regulated mycotoxins and related metabolites (such as DON, NIV, ZEN, α-ZEL, FBs, EAs) as well as emerging mycotoxins from *Fusarium* spp. (ENN, BEA, CUL), *Alternaria* spp. (TeA, AHO, AME) and *Aspergillus* (STC) along with *Penicillium* toxins (e.g. MA, ROQ C) and other less-studied metabolites. Specifically, the group of *Fusarium* spp. mycotoxins was the second most abundant, especially in MS having almost 8 times higher mean concentration compared to that of GS. One MS sample surpassed the maximum concentration of *Penicillium* spp. metabolites. *Fusarium-*derived mycotoxins such as DON, NIV, ZEN and ENN B are commonly found in whole-plant maize, pastures, and their silages (Gruber-Dorninger et al. 2017; Panasiuk et al. 2019; Reisinger et al. 2019; Vandicke et al. 2019). The levels of several fusarial mycotoxins (e.g. DON, NIV, ZEN, FB1, FB2, 15-hydroxyculmorin, culmorin, ENNs, equisetin, MAS and HT-2 toxin) found in mouldy MS in the current study in Austria were still below the maximum values reported in 158 MS samples (not specifically mouldy hot spots) from ten European countries (Reisinger et al. 2019). However, in our study, fusaric acid (FA) was found in high concentrations in the mouldy spots, especially in the two samples contaminated with *F. verticillioides* (precisely with 1.00 × 10^7^ CFU/g and 5.00 × 10^6^ CFU/g and respective concentrations of 408,000 µg/kg and 7,790 µg/kg)*,* like previous reports (Brown et al. 2012; Merel et al. 2020). This could suggest that some fusarial potentially toxic metabolites such as FA could be produced during ensiling by *Fusarium* spp. (Wambacq et al. 2016). Interestingly, FA can enhance the activity of other *Fusarium* mycotoxins such as moniliformin, trichothecenes and fumonisins (Bacon et al. 1996; D'Mello et al. 1999). Additionally, its antimicrobial activity against *Ruminococcus albus* and *Methanobrevibacter ruminantium* has been described (May et al. 2000), possibly impacting the functionality of the rumen microbiome. AFs, OTA and T2 were not present in any sample, which was in accordance with previous European reports in non-mouldy silage (Driehuis et al. 2008a, b; Zachariasova et al. 2014; Panasiuk et al. 2019). Our study found a high occurrence of emerging fusarial mycotoxins such as ENNs and BEA, in line with the results reported by McElhinney et al. (2016). One of the most studied mycotoxin combinations is DON-ZEN, which was detected in our study with a frequency of 61% in mouldy MS, similar to a previous European survey on non-mouldy MS (Reisinger et al. 2019). Considerably high occurrences of DON-ZEN co-contamination in MS and dairy diets have been reported by other authors (Kosicki et al. 2016; Panasiuk et al. 2019). Several studies proposed that MS is a major source of DON and ZEN in dairy feeds (Driehuis et al. 2008a, b; Panasiuk et al. 2019; Rodríguez-Blanco et al. 2019). Vandicke et al. (2021) proposed that, at the first phase of the ensiling process, the levels of mycotoxins such as parent forms could decline by elution, degradation, and absorption (caused by lactic acid bacteria). Subsequently, during the stable phase, under aerobic conditions (silos that are not properly sealed off) silage can be colonized by fungi again, producing additional mycotoxins, such as Afs, FBs, DON, ZEN and related metabolites. While the presence of field, fungi like *Fusarium and Alternaria* could become less significant in ensiled material as shown by Mansfield and Kuldau (2007) and the present study, our data further indicate that their metabolites may persist longer in the ensiled material. Still, available information about the effect of the ensiling on the fate of *Fusarium* spp. mycotoxins suggests a possible reduction in levels ZEN, DON and FBs after fermentation is contradictory (Richter et al. 2002; Boudra and Morgavi 2008; Vandicke et al. 2021), while other reports showed that the contamination levels remain unchanged (González Pereyra et al. 2014) or even increase (González Pereyra et al. 2008). Jensen et al. (2020) studied the fate of DON and ZEN as well as their modified forms using laboratory-scale silos. Comparing the concentration of mycotoxins before and after ensilage, they found that the levels of ZEN, α-ZOL, β-ZOL and ZEN-4-sulphate were constant, but the concentrations of DON increased significantly, whereas the levels of DON-3-glucoside and acetylated forms decreased proportionally. Additionally, to study the production of fungal secondary metabolites and their influencing/associated factors, controlled experimental approaches are needed. Studies under controlled environmental and ensiling conditions would reduce the external variation introduced by different locations, geo-climatic conditions, crop varieties, agricultural practices (e.g. use of fertilizers and fungicides) and other factors that influence the mycotoxins synthesis.

As found in previous studies, our results evidenced significantly higher levels of contamination with total fungal metabolites, specifically those produced mainly by Fusaria and Penicillia as well as EAs in MS compared to GS (Driehuis et al. 2008a, b; Panasiuk et al. 2019; Venslovas et al. 2021). In agreement with a recent study carried out in Germany (Dänicke et al. 2020), we also verified that mouldy spots of MS showed a broader spectrum of mycotoxins compared to GS. It has been described those high levels of water-soluble carbohydrates promote the growth of *P. roqueforti* (Pitt et al. 1991). Likewise, starch induces trichothecene production in *F. graminearum* (Oh et al. 2016). Thus, the higher content of water-soluble carbohydrates including starch found in maize plants in comparison with grasses, legumes and their mixtures could explain the higher levels of mycotoxins and other metabolites.

Regarding metabolites derived mainly from *Aspergillus* spp., although the strictly regulated aflatoxin B_1_ and other AFs were not found, their precursors averufin and STC were detected in both mouldy silages. The latter, STC is a carcinogen compound and has been associated with immunotoxin and immunomodulatory activity, together with mutagenic effects, which justifies its toxicological interest (EFSA 2013; Viegas et al. 2020). The levels of STC found recently in pastures from Austria and in European MS presented a maximum concentration below 10 µg/kg (Reisinger et al. 2019), whereas the mouldy spots of GS and MS here studied here presented maximum levels of 26.6 and 4.75, respectively. It has been suggested that STC can be produced pre-and post-harvest (Mo et al. 2015). In general, the information available on exposure data of dairy cows to the mentioned precursors of AF is scarce (EFSA 2013; Gruber-Dorninger et al. 2017). Concerning detected emerging *Alternaria* mycotoxins, TeA, AOH and AME are considered to have toxicological relevance (Solfrizzo 2017). Regarding toxicity, the most important mycotoxin produced by *Alternaria* spp. is TeA (Kumari and Tirkey 2019), which targets protein synthesis inhibition at the ribosomal level, while the benzopyrene derivatives AOH and AME, known for their genotoxic effects (Gil-Serna et al. 2014), also showed strong synergistic estrogenic effects in combination with the fusarial mycoestrogen ZEN even at very low concentrations (Vejdovszky et al. 2017). In our study, levels of TeA in mouldy GS (range: 195 µg/kg -1920 µg/kg) and MS (range: 57.2 µg/kg -7,270 µg/kg) were considerably higher than levels found in ensiled maize from several European countries (maximum: 727 µg/kg) (Reisinger et al. 2019). *Alternaria*-derived toxins (AOH, AME and TeA) can be produced on-field and post-harvest. Contamination with *Alternaria* metabolites has been detected in pastures and maize (Nichea et al. 2015; Reisinger et al. 2019; Penagos-Tabares et al. 2021), and their production has been described during ensiling (Dacero et al. 1997). In our case, the relatively low levels of AOH and AME in mouldy silages (< 50 µg/kg) seem to indicate that these metabolites are not major products produced during ensiling or in mouldy spots, fitting with the findings of Storm et al. (2014). The current results emphasize the role of TeA as the most abundant mycotoxins produced by *Alternaria* spp. in mouldy spots of silages (median concentration: 569 µg/kg in GS and 275 µg/kg in MS), while it was not detected in pastures (Penagos-Tabares et al. 2021). This may indicate that this mycoestrogen could be produced post-harvest in mouldy spots. Furthermore, the information is still scarce regarding the occurrence and toxic effects of *Alternaria*-derived toxins in animals, and therefore, health risks associated with these toxins in feeds have not yet been clarified (EFSA 2011).

Fungal biomass, DNA and colony counts are not directly associated with mycotoxin production, and there is not essentially a direct association between the presence of fungal species and the levels of mycotoxins in silage sampled at a certain point of time (Barug et al. 2006; Magan 2006; Storm et al. 2008). However, there is emerging evidence that they could be able to predict the presence of some mycotoxins (Cheli et al. 2013). Except for *P. roqueforti* and *Penicillium* metabolites in mouldy MS, our study found generally no correlation between mould counts and corresponding metabolites detected. The increased counts of *P. roqueforti* are closely related to superior levels of total *Penicillium*-derived metabolites (Supplementary Figure S2a), fitting with the results of Auerbach et al. (1998), which indicated that the *P. roqueforti* counts can be utilized as a criterion to predict the grade of contamination with toxins like ROQ C produced by this mould. In addition, these researchers emphasized that the feeding of silages with mouldy counts > 10^6^ CFU/g should be stringently avoided of dietary rations of farm animals due to the possibility of contamination with *P. roqueforti*-toxins (Auerbach et al. 1998). Moreover, other studies seem to indicate that ROC C has a positive correlation with fungal growth because this secondary metabolite is produced by some fungi as a transportable extracellular nitrogen reserve (Boichenko et al. 2002; Wambacq 2017). However, a recent study analysed the presence of *Fusarium* mycotoxins in MS from seed to feed and found no correlations between fungal DNA and mycotoxin concentrations (Vandicke et al. 2021). Therefore, a simple investigation of microbial population is not always a good indicator of contamination with the most relevant regulated mycotoxins (AFs, OTA, ZEN, FBs, and DON) (Schmidt et al. 2015; Carvalho et al. 2016), which is in accordance with our results.

Additionally, it is important to remark that traditional and routinary techniques for the determination of mycobiota in feedstuffs by dilution and plating used in the present study, as well as in other studies (Baggerman 1981; Skaar and Stenwig 1996; O'Brien et al. 2005; Richard et al. 2007; Schenck et al. 2019). Although dominant and typical mycobiota responsible for the deterioration of silages such as *Penicillium spp.*, *Aspergillus* spp. and yeasts could be cultivated and identified (Mansfield and Kuldau 2007), selective media may not indicate with absolute certainty a complete profile of the mycobiota in the field or silage (Storm et al. 2008). The use of suitable and diversified culture conditions (different media and incubation in a modified atmosphere) may expand the picture of the silage’s microbiota. Thus, the development of standardized methods has been strongly suggested (Storm et al. 2008). Furthermore, molecular approaches could provide a more complete picture of the microbial ecology of ensiling, aerobic deterioration, and subsequently a more accurate taxonomical identification (McAllister et al. 2018). For instance, Mansfield and Kuldau (2007) showed that a molecular approach using DNA sequences detected a greater number of fungal species than microbiological evaluation with selective media and morphological identification. For instance, *Alternaria* spp. were only detected with the molecular analysis. Also, considering the heterogeneity of mycotoxins in silages (McElhinney et al. 2016), interpretation and extrapolation of our findings may be limited to dominant mould species that colonize the superficial surfaces of certain kinds of silages.

The mouldy spots of silages investigated in this study were found to harbour several opportunist pathogens such as *A. fumigatus*, *M. circinelloides, Rhizomucor* spp.*, Lichtheimia* spp. and *P. boydii*, pointing out an additional concern regarding the health risks for livestock and humans who are exposed to mouldy silages. These pathogenic moulds are relevant epidemiologically as causative agents of respiratory infectious diseases (mycosis), representing a higher health risk to animals and humans (farmworkers) (Alonso et al. 2013; de Hoog et al. 2020; Eucker et al. 2001; Pal et al. 2013). Mouldy silages could also contribute to a form of hypersensitivity pneumonitis denominated farmer’s lung disease (Wuhrmann et al. 1965; Cano-Jiménez et al. 2016; Barnes et al. 2021) and possible cases of acute intoxications (mycotoxicosis) in workers handling high contaminated mouldy silage cannot be discarded (Emanuel et al. 1975; Gordon et al. 1993). Silages are economically relevant forage sources in dairy production, but they also represent sources of mycotoxin mixtures due to mould proliferation. Considering that spoilage of silage is heterogeneous and mouldy spots are not always visually detectable, the most important preventive measures thus consist of improving the storage conditions and sensibilization of farmworkers for the utilization of the respiratory protective equipment to avoid the inhalation of fungal organisms with pathogenic potential or their antigens (Cano-Jiménez et al. 2016).

This pilot study provides insight into the most occurrent fungal species spoiling GS and MS in Austria, confirming the previously called status of *P. roqueforti* as the “silage mould”. The co-occurrence of other toxigenic along with non-toxigenic fungal organisms, some of them opportunistic pathogens of animals and humans was corroborated. Data on the profiles of mycotoxins and other metabolites contained in mouldy silages demonstrated high concentrations of *Penicillium*-derived compounds and a considerable amount of wide spectrum regulated, emerging, modified and less known (potential) mycotoxins. The routinary fungal counts and the levels of (toxic) secondary metabolites in mouldy silages were not correlated, with exception of *P. roqueforti’s* counts and some metabolites derived from *Penicillium* spp. in MS. Several pre-and post-harvest fungal toxins were detected in higher levels in MS compared to GS, suggesting that GS could be a better option as a source of animal feed in terms of lower mycotoxigenic risk. Further research focused on the occurrence, dietary levels and toxicity of mouldy silage-derived compounds, and their effects on the rumen microbiota, “mouldy silage syndrome” and carry-over via milk are needed. Diagnostics, prevention and remediation strategies for reducing at minimum the mould growth and mycotoxin production in ensiled feeds as well as the influencing environmental factors must be further investigated.

## Supplementary information

**Supplementary Figure S1.** Co-occurrence of fungal species isolated from mouldy spots of (a) grass and (b) maize silage.

**Supplementary Figure S2.** Linear regressions and bar diagram showing significant associations detected in the analysed mouldy silages. (a) Relationship between counts of Penicillium roqueforti and levels of total Penicillium metabolites. (b) Association between levels of total Penicillium metabolites as well as (c) of andrastins A, B and C, roquefortines C and D with the total concentration of fungal metabolites detected in mouldy spots of grass silage. (d) Associations between total Penicillium metabolites and specific metabolites (AND A, AND B and ROC D) in mouldy maize silages. (e) Relationship between Aspergillus-derived metabolites and unspecific metabolites in mouldy spots of maize silage. (f) Relationship between zearalenone and deoxynivalenol in mouldy spots of maize. (g) Correlation of festuclavine with the Penicillium derived metabolites (AND A, ROQ C and ROC D) in mouldy grass silage. Sy.x = Standard error of estimate. Significance level at p-value < 0.05).

Below is the link to the electronic supplementary material.

Supplementary file1 (DOCX 797 KB)

## Data Availability

Data transparency.

## Code availability

Software application or custom code.
