# Supplementary material for: Fungal species and mycotoxins in mouldy spots of grass and maize silages in Austria
Source: Mycotoxin Res. 2022 Mar 26;38(2):117–36. doi: 10.1007/s12550-022-00453-3 (PMC9038934; doi:10.1007/s12550-022-00453-3)
Supplement: Supplementary file 1 — Supplementary file1 (DOCX 797 KB) [file 12550_2022_453_MOESM1_ESM.docx]

**Supplementary Table S1.** Occurrences and counts (CFU/g) of fungal cultivated fungal species detected in spots of mouldy grass and maize silages

| **Fungal species** | **Grass silage (n=19)** | | | | | | | | | | **Maize silage (n=28)** | | | | | | | | | | | **Mann-Whitney Test** | |
| --- | --- | --- | --- | --- | --- | --- | --- | --- | --- | --- | --- | --- | --- | --- | --- | --- | --- | --- | --- | --- | --- | --- | --- |
|  | **Positive Samples [%]** | **Count (CFU/g)** | | | | | | | | |  | **Positive Samples [%]** | **Counts (CFU/g)** | | | | | | | | |  |  |
|  |  | **Average ± SD** | | |  | **Median** |  | **Range** | | |  |  | **Average ± SD** | | |  | **Median** |  | **Range** | | | ***p*-value** | |
| ***Acremonium* spp.** | 5 |  |  |  |  |  |  | 1.0 × 10^4^ | | |  | 0 |  |  |  |  |  |  |  | | |  |  |
| ***Aspergillus fumigatus*** | 26 | 6.0 × 10^5^ | ± | 4 6 × 10^5^ |  | 6.0 × 10^5^ |  | 2.0 × 10^5^ | - | 1.0 × 10^6^ |  | 29 | 6.6 × 10^6^ | ± | 4.3 × 10^6^ |  | 1.00 × 10^7^ |  | 1.0 × 10^6^ | - | 1.0 × 10^7^ | 0.688 |  |
| ***Fusarium* spp.** | 0 |  |  |  |  |  |  |  | | |  | 4 |  |  |  |  |  |  | 1.0 × 10^5^ | | |  |  |
| ***Fusarium verticillioides*** | 0 |  |  |  |  |  |  |  | | |  | 7 | 7.5 × 10^6^ | ± | 3.5 × 10^6^ |  | 7.5 × 10^6^ |  | 5.0 × 10^6^ | - | 1.0 × 10^7^ |  |  |
| ***Geotrichum candidum*** | 26 | 4.6 × 10^5^ | ± | 3.4 × 10^5^ |  | 4.0 × 10^5^ |  | 1.0 × 10^5^ | - | 1.0 × 10^6^ |  | 46 | 1.70× 10^6^ | ± | 2.8 × 10^6^ |  | 5.5 × 10^5^ |  | 6.0 × 10^5^ | - | 1.0 × 10^7^ | 0.142 |  |
| ***Hypopichia burtonii*** | 5 |  |  |  |  |  |  | 1.0 × 10^6^ | | |  | 0 |  |  |  |  |  |  |  | | |  |  |
| ***Lichtheimia corymbifera*** | 16 | 1.7 × 10^6^ | ± | 2.0 × 10^6^ |  | 1.0 × 10^6^ |  | 1.0 × 10^5^ | - | 4.0 × 10^6^ |  | 14 | 3.95 × 10^6^ | ± | 4.6 × 10^6^ |  | 2.8 × 10^6^ |  | 3.0 × 10^5^ | - | 1.0 × 10^7^ | 0.946 |  |
| ***Monascus ruber*** | 37 | 3.5 × 10^6^ | ± | 3.5 × 10^6^ |  | 3.0 × 10^6^ |  | 1.0 × 10^5^ | - | 1.0 × 10^7^ |  | 29 | 3.9 × 10^6^ | ± | 4.1 × 10^6^ |  | 2.5 × 10^6^ |  | 1.0 × 10^5^ | - | 1.0 × 10^7^ | 0.591 |  |
| ***Mucor circinelloides*** | 16 | 4.4 × 10^6^ |  | 5.1 × 10^6^ |  | 3.0 × 10^6^ |  | 1.0 × 10^5^ | - | 1.0 × 10^7^ |  | 25 | 2.5 × 10^6^ | ± | 3.7 × 10^6^ |  | 6.0 × 10^5^ |  | 4.0 × 10^5^ | - | 1.0 × 10^7^ | 0.548 |  |
| ***Paecilomyces niveus*** | 16 | 7.0 × 10^5^ | ± | 3.6 × 10^5^ |  | 8.0 × 10^5^ |  | 3.0 × 10^5^ | - | 1.0 × 10^6^ |  | 36 | 4.2 × 10^6^ | ± | 4.2 × 10^6^ |  | 2.3 × 10^6^ |  | 2.0 × 10^5^ | - | 1.0 × 10^7^ | 0.103 |  |
| ***Paecilomyces variotii*** | 0 |  |  |  |  |  |  |  | | |  | 4 |  |  |  |  |  |  | 5.0 × 10^5^ | | |  |  |
| ***Penicillium roqueforti*** | 74 | 1.5 × 10^6^ | ± | 2.8 × 10^6^ |  | 2.0 × 10^6^ |  | 1.0 × 10^4^ | - | 1.0 × 10^7^ |  | 71 | 4.9 × 10^6^ | ± | 4.5 × 10^6^ |  | 4.0 × 10^6^ |  | 2.0 × 10^5^ | - | 1.0 × 10^7^ | 0.1097 |  |
| ***Pseudallescheria boydii*** | 5 |  |  |  |  |  |  | 1.0 × 10^4^ | | |  | 14 | 2.4 × 10^6^ | ± | 2.7 × 10^6^ |  | 2.3 × 10^6^ |  | 1.0× 10^5^ | - | 5.0 × 10^6^ |  |  |
| ***Rhizomucor pusillus*** | 0 |  |  |  |  |  |  |  | | |  | 7 | 5.5 × 10^5^ | ± | 6.4 × 10^5^ |  | 5.5 × 10^5^ |  | 1.0 × 10^5^ | - | 1.0 × 10^6^ |  |  |
| ***Saccharomyces* spp.** | 47 | 1.6 × 10^6^ | ± | 1.9 × 10^6^ |  | 1.0 × 10^6^ |  | 1.0 × 10^5^ | - | 5.0 × 10^6^ |  | 43 | 3.8 × 10^6^ | ± | 4.1 × 10^6^ |  | 2.0 × 10^6^ |  | 2.0 × 10^4^ | - | 1.0 × 10^7^ | 0.967 |  |
| ***Scopulariopsis brevicaulis*** | 11 | 6.0 × 10^5^ | ± | 5.7 × 10^5^ |  | 6.0 × 10^5^ |  | 2.0 × 10^5^ | - | 1.0 × 10^6^ |  | 0 |  |  |  |  |  |  |  | | |  |  |
| ***Verticillium* spp.** | 0 |  |  |  |  |  |  |  | | |  | 4 |  |  |  |  |  |  | 5.00 × 10^5^ | | |  |  |
| **Sum of moulds** | 100 | 3.7 ×10^6^ | ± | 4.9 × 10^5^ |  | 1.4 × 10^6^ |  | 1.0 × 10^4^ | - | 1.5 × 10^7^ |  | 100 | 1.0 × 10^7^ | ± | 5.51 × 10^6^ |  | 1.05 × 10^7^ |  | 1.00 × 10^6^ | - | 2.1 × 10^7^ | <0.001 | * |
| **Sum of yeasts** | 68 | 1.4 ×10^6^ | ± | 1.7 × 10^5^ |  | 1.0 × 10^6^ |  | 1.0 × 10^5^ | - | 5.0 × 10^6^ |  | 75 | 3.2 × 10^6^ | ± | 3.80 × 10^6^ |  | 1.50 × 10^6^ |  | 5.00 × 10^4^ | - | 1.1 × 10^7^ | 0.363 |  |
| **Total fungi**  **(Yeasts and moulds)** | 100 | 4.6 ×10^6^ | ± | 5.0 × 10^6^ |  | 3.1 × 10^6^ |  | 1.0 × 10^4^ | - | 1.5 × 10^7^ |  | 100 | 1.3 × 10^7^ | ± | 6.50 × 10^6^ |  | 1.14 × 10^7^ |  | 2.50 × 10^6^ | - | 2.2 × 10^7^ | <0.001 | * |

* Significantly different (*p*-value < 0.05)

**Supplementary Table S2.** Occurrences and levels of mycotoxins and other metabolites detected in spots of mouldy grass and maize silages

|  |  | **Grass silage (n=19)** | | | | | | | | | |  | **Maize silage (n=28)** | | | | | | | | **Mann-Whitney Test** | |
| --- | --- | --- | --- | --- | --- | --- | --- | --- | --- | --- | --- | --- | --- | --- | --- | --- | --- | --- | --- | --- | --- | --- |
| **Group** | **Metabolites** | **Positive Samples (%)^1^** | **Concentration^2^** | | | | | | | | |  | **Positive Samples (%)^1^** | **Concentration^2^** | | | | | | | ***p*-value** | |
|  |  |  | **Average ± SD** | | | **Median** | | | **Range** | | |  |  | **Average ± SD** | | | **Median** | **Range** | | |  |  |
| **Ergot alkaloid** | **Agroclavine** | 11 | 2.47 | ± | 0.3 | 2.47 | | | 2.25 | - | 2.68 |  | 32 | 8.06 | ± | 7.35 | 6.43 | 1.44 | - | 23.1 | 0.079 |  |
|  | **Chanoclavine** | 58 | 36.5 | ± | 73.6 | 5.78 | | | 0.16 | - | 225 |  | 54 | 160 | ± | 445 | 18.7 | 0.17 | - | 1,740 | 0.778 |  |
|  | **Festuclavine*** | 63 | 63.7 | ± | 123 | 14.9 | | | 0.35 | - | 435 |  | 82 | 313 | ± | 444 | 86.9 | 1.07 | - | 1,360 | 0.026 | * |
| ***Alternaria* spp.** | **Alternariol** | 16 | 10.2 | ± | 12.2 | 4.35 | | | 2.09 | - | 24.2 |  | 29 | 2.34 | ± | 3.36 | 1.14 | 0.3 | - | 10.4 | 0.482 |  |
|  | **Alternariolmethylether** | 26 | 4.13 | ± | 2.3 | 3.5 | | | 1.6 | - | 7.32 |  | 29 | 1.78 | ± | 1.62 | 1.31 | 0.13 | - | 4.71 | 0.857 |  |
|  | **Altersetin** | 32 | 176 | ± | 315 | 58.1 | | | 5.13 | - | 818 |  | 36 | 13.6 | ± | 9.85 | 12.6 | 1.11 | - | 31.6 | 0.792 |  |
|  | **Infectopyron** | 5 |  | | | | | 26.2 | | | |  | 21 | 32.1 | ± | 21.7 | 22 | 11 | - | 66.1 | 0.183 |  |
|  | **Tenuazonic acid** | 53 | 781 | ± | 552 | 569 | | | 195 | - | 1,920 |  | 61 | 785 | ± | 1,720 | 275 | 57.2 | - | 7,270 | 0.651 |  |
| ***Aspergillus* spp.** | **Averufin** | 21 | 2.75 | ± | 2.64 | 2.08 | | | 0.34 | - | 6.51 |  | 7 | 2.01 | ± | 0.78 | 2.01 | 1.46 | - | 2.56 | 0.15 |  |
|  | **Bis(methylthio)gliotoxin** | 11 | 133 | ± | 184 | 133 | | | 2.19 | - | 263 |  | 32 | 152 | ± | 242 | 63.8 | 6.53 | - | 756 | 0.088 |  |
|  | **Chaetominine*** | 5 |  | | | | 439 | | | | |  | 32 | 468 | ± | 431 | 370 | 10.5 | - | 1,430 | 0.037 | * |
|  | **Demethylsulochrin** |  |  |  |  |  | | |  |  |  |  | 14 | 129 | ± | 113 | 85.3 | 48.6 | - | 296 | 0.136 |  |
|  | **Fumagillin** |  |  |  |  |  | | |  |  |  |  | 14 | 2,190 | ± | 2,800 | 1,230 | 0.4 | - | 6,280 | 0.136 |  |
|  | **Fumigaclavine** | 26 | 276 | ± | 557 | 5.34 | | | 1.56 | - | 1,270 |  | 32 | 563 | ± | 729 | 212 | 1.08 | - | 2,040 | 0.544 |  |
|  | **Fumigaclavine C** | 37 | 1,800 | ± | 4,000 | 81.3 | | | 11.3 | - | 10,780 |  | 36 | 3,950 | ± | 7,430 | 857 | 5.61 | - | 23,300 | 0.948 |  |
|  | **Fumiquinazoline A** | 11 | 55.5 | ± | 52.9 | 55.5 | | | 18.1 | - | 92.9 |  | 25 | 520 | ± | 1,110 | 62.7 | 21 | - | 3,023 | 0.182 |  |
|  | **Fumiquinazoline D** | 26 | 433 | ± | 879 | 10.5 | | | 1.77 | - | 2,000 |  | 32 | 3,890 | ± | 9,230 | 1,080 | 1.66 | - | 28,400 | 0.514 |  |
|  | **Fumitremorgin C** | 5 |  |  |  |  | | | 33.9 | | |  | 21 | 169 | ± | 242 | 58.9 | 26.3 | - | 651 | 0.134 |  |
|  | **Gliotoxin** | 5 |  |  |  |  | | | 79.3 | | |  | 14 | 47 | ± | 46.6 | 46.8 | 5.09 | - | 89.2 | 0.39 |  |
|  | **Helvolic acid** | 5 |  |  |  |  | | | 131 | | |  | 18 | 406 | ± | 703 | 76.4 | 53.4 | - | 1,660 | 0.32 |  |
|  | **Kojic acid** | 21 | 63.2 | ± | 44.5 | 43.5 | | | 36.2 | - | 129 |  | 43 | 97.7 | ± | 103 | 54.3 | 16.1 | - | 353 | 0.136 |  |
|  | **Methylsulochrin** | 26 | 541 | ± | 1,060 | 10.9 | | | 1.98 | - | 2,420 |  | 43 | 797 | ± | 1,620 | 57.1 | 0.78 | - | 4,910 | 0.265 |  |
|  | **Mevinolin** | 53 | 516 | ± | 611 | 206 | | | 3.26 | - | 1,520 |  | 25 | 550 | ± | 740 | 185 | 4.03 | - | 2,050 | 0.065 |  |
|  | **Pinselin*** | 5 |  |  |  |  | | | 1.29 | | |  | 43 | 116 | ± | 173 | 48.5 | 1.91 | - | 594 | 0.003 | * |
|  | **Pseurotin A** | 5 |  |  |  |  | | | 1,440 | | |  | 25 | 1,550 | ± | 2,560 | 247 | 20.6 | - | 7,100 | 0.101 |  |
|  | **Pyripyropene A** | 5 |  |  |  |  | | | 108 | | |  | 21 | 197 | ± | 315 | 53 | 17.6 | - | 824 | 0.183 |  |
|  | **Sphingofungin B** |  |  |  |  |  | | |  |  |  |  | 11 | 7,250 | ± | 9,670 | 3,280 | 206 | - | 18,300 | 0.262 |  |
|  | **Sphingofungin D** | 5 |  |  |  |  | | | 5.29 | | |  | 14 | 195 | ± | 313 | 58 | 4.39 | - | 658 | 0.292 |  |
|  | **Sterigmatocystin*** | 37 | 6.89 | ± | 9.79 | 1.3 | | | 0.09 | - | 26.6 |  | 7 | 2.49 | ± | 3.2 | 2.49 | 0.23 | - | 4.75 | 0.011 | * |
|  | **Trypacidin** | 42 | 120 | ± | 290 | 2.88 | | | 0.91 | - | 833 |  | 21 | 451 | ± | 696 | 103 | 0.85 | - | 1,750 | 0.21 |  |
|  | **Versicolorin C** | 5 |  |  |  |  | | | 7 | | |  | 4 |  |  |  |  | 4.03 | | | 0.754 |  |

^1^Samples with values > limit of detection (LOD); ^2^Excluding data < LOD. In case values >LOD and < limit of quantification (LOQ), LOQ/2 was used for calculation.

* Significantly different (p-value < 0.05)

**Supplementary Table S2. Cont.** Occurrences and levels of mycotoxins and other metabolites detected in spots of mouldy grass and maize silages

|  |  | **Grass silage (n=19)** | | | | | | | |  | **Maize silage (n=28)** | | | | | | | | **Mann-Whitney Test** | |
| --- | --- | --- | --- | --- | --- | --- | --- | --- | --- | --- | --- | --- | --- | --- | --- | --- | --- | --- | --- | --- |
| **Group** | **Metabolites** | **Positive Samples (%)^1^** | **Concentration^2^** | | | | | | |  | **Positive Samples (%)^1^** | **Concentration^2^** | | | | | | | ***p*-value** | |
|  |  |  | **Average ± SD** | | | **Median** | **Range** | | |  |  | **Average ± SD** | | | **Median** | **Range** | | |  |  |
| ***Fusarium* spp.** | **15-Hydroxyculmorin** | 5 |  |  |  |  | 16.3 | | |  | 46 | 143 | ± | 192 | 76.5 | 33.7 | - | 742 | 0.001 | * |
|  | **alpha-Zearalenol** | 0 |  |  |  |  |  |  |  |  | 11 | 62.1 | ± | 56.3 | 61.4 | 6.08 | - | 119 | 0.262 |  |
|  | **Antibiotic Y** | 16 | 119 | ± | 196 | 9.54 | 3.58 | - | 345 |  | 7 | 293 | ± | 403 | 293 | 7.93 | - | 578 | 0.59 |  |
|  | **Apicidin** | 5 | 7.92 |  |  |  |  |  |  |  | 71 | 23.8 | ± | 25.4 | 17.2 | 3.81 | - | 111 | <0.001 | * |
|  | **Aurofusarin** | 32 | 35.5 | ± | 23.2 | 41.2 | 4.07 | - | 59.9 |  | 75 | 61.8 | ± | 50.5 | 41 | 3.92 | - | 171 | 0.004 | * |
|  | **Beauvericin** | 47 | 19.7 | ± | 40.4 | 1.83 | 0.2 | - | 125 |  | 86 | 30.1 | ± | 36.7 | 17.7 | 3.93 | - | 153 | <0.001 | * |
|  | **Bikaverin** | 0 |  |  |  |  |  |  |  |  | 46 | 9.7 | ± | 5.68 | 7.04 | 3.53 | - | 22.7 | <0.001 | * |
|  | **Butenolide** | 0 |  |  |  |  |  |  |  |  | 14 | 15.2 | ± | 6.6 | 15.4 | 7.07 | - | 23.1 | 0.136 |  |
|  | **Chrysogine** | 53 | 34.4 | ± | 31.8 | 23.1 | 4.61 | - | 102 |  | 29 | 6.64 | ± | 5.07 | 4.44 | 2.35 | - | 15.8 | 0.018 | * |
|  | **Culmorin** | 42 | 83.8 | ± | 65.1 | 62.7 | 5.77 | - | 179 |  | 79 | 302 | ± | 366 | 199 | 20.7 | - | 1,360 | <0.001 | * |
|  | **Deoxynivalenol** | 16 | 19.6 | ± | 10.2 | 20 | 9.24 | - | 29.6 |  | 79 | 291 | ± | 285 | 224 | 30 | - | 1,220 | <0.001 | * |
|  | **Enniatin A** | 37 | 1.36 | ± | 1.75 | 0.81 | 0.02 | - | 4.9 |  | 43 | 0.85 | ± | 0.81 | 0.67 | 0.01 | - | 2.17 | 0.74 |  |
|  | **Enniatin A1** | 58 | 3.89 | ± | 6.01 | 2.2 | 0.17 | - | 20.3 |  | 75 | 10.9 | ± | 14.3 | 4.27 | 0.2 | - | 51.4 | 0.041 | * |
|  | **Enniatin B** | 84 | 11.1 | ± | 13.3 | 6.37 | 0.27 | - | 44.5 |  | 86 | 8.26 | ± | 10.5 | 4.94 | 0.11 | - | 44.7 | 0.572 |  |
|  | **Enniatin B1** | 68 | 12.6 | ± | 21.1 | 7.19 | 0.64 | - | 80.7 |  | 68 | 19.4 | ± | 26.8 | 7.16 | 0.05 | - | 95.3 | 0.887 |  |
|  | **Enniatin B2** | 26 | 0.7 | ± | 0.79 | 0.44 | 0.14 | - | 2.08 |  | 32 | 0.46 | ± | 0.32 | 0.42 | 0.11 | - | 1.06 | 0.719 |  |
|  | **Epiequisetin** | 37 | 7.15 | ± | 8.45 | 2.65 | 1.01 | - | 22.3 |  | 46 | 6.42 | ± | 6.95 | 3.45 | 0.3 | - | 23.5 | 0.559 |  |
|  | **Equisetin** | 47 | 39.4 | ± | 67.1 | 8.55 | 0.65 | - | 181 |  | 46 | 9.16 | ± | 11.2 | 4.12 | 1.23 | - | 41.9 | 0.771 |  |
|  | **Fumonisin B1** | 0 |  |  |  |  |  |  |  |  | 75 | 88.4 | ± | 79.0 | 58.8 | 14 | - | 356 | <0.001 | * |
|  | **Fumonisin B2** | 0 |  |  |  |  |  |  |  |  | 50 | 28.7 | ± | 22.4 | 25.6 | 10.1 | - | 97.8 | <0.001 | * |
|  | **Fumonisin B3** | 0 |  |  |  |  |  |  |  |  | 11 | 20.9 | ± | 3.2 | 21.1 | 17.6 | - | 24 | 0.262 |  |
|  | **Fumonisin B4** | 0 |  |  |  |  |  |  |  |  | 11 | 10.7 | ± | 4.68 | 12.8 | 5.39 | - | 14.1 | 0.262 |  |
|  | **Fusaric acid** | 0 |  |  |  |  |  |  |  |  | 18 | 83,300 | ± | 182,000 | 217 | 108 | - | 408,000 | 0.1 |  |
|  | **Fusarin C** | 5 |  |  |  |  | 599 | | |  | 4 |  | | |  | 166 | | | 0.754 |  |
|  | **Fusarinolic acid** | 0 |  |  |  |  |  |  |  |  | 11 | 11,180 | ± | 19,210 | 85.5 | 84.4 | - | 33,400 | 0.262 |  |
|  | **Gibepyron D** | 5 |  |  |  |  | 1,563 | | |  | 0 |  |  |  |  |  |  |  | 0.404 |  |
|  | **HT-2 toxin** | 0 |  |  |  |  |  |  |  |  | 21 | 16.8 | ± | 9.65 | 14.6 | 4.81 | - | 31 | 0.068 |  |
|  | **Moniliformin** | 5 |  |  |  |  | 8.47 | | |  | 29 | 5.76 | ± | 5.06 | 4.46 | 1.56 | - | 17 | 0.072 |  |
|  | **Monoacetoxyscirpenol** | 0 |  |  |  |  |  |  |  |  | 4 |  | | |  | 6.01 | | | >0.999 |  |
|  | **Nivalenol** | 5 | 36 |  |  |  |  |  |  |  | 89 | 281 | ± | 219 | 191.1 | 38.9 | - | 852 | <0.001 | * |
|  | **Siccanol** | 47 | 8,130 | ± | 20,700 | 1,400 | 200 | - | 63,200 |  | 82 | 3,200 | ± | 5,380 | 1,580 | 154 | - | 26,100 | 0.015 | * |
|  | **Zearalenone** | 21 | 178 | ± | 327 | 20.2 | 3.43 | - | 668 |  | 61 | 15 | ± | 14.4 | 10.6 | 2.08 | - | 53.9 | 0.016 | * |

^1^Samples with values > limit of detection (LOD); ^2^Excluding data < LODIn case values >LOD and < limit of quantification (LOQ), LOQ/2 was used for calculation.

* Significantly different (p-value < 0.05)

**Supplementary. Table S2. Cont.** Occurrences and levels of mycotoxins and other metabolites detected in spots of mouldy grass and maize silages

|  |  | **Grass silage (n=19)** | | | | | | | |  | **Maize silage (n=28)** | | | | | | | | **Mann-Whitney Test** | |
| --- | --- | --- | --- | --- | --- | --- | --- | --- | --- | --- | --- | --- | --- | --- | --- | --- | --- | --- | --- | --- |
| **Group** | **Metabolite** | **Positive Samples (%)^1^** | **Concentration^2^** | | | | | | |  | **Positive Samples (%)^1^** | **Concentration^2^** | | | | | | | ***p*-value** | |
|  |  |  | **Average ± SD** | | | **Median** | **Range** | | |  |  | **Average ± SD** | | | **Median** | **Range** | | |  |  |
| **Other fungi** | **Emindole SA** |  |  |  |  |  |  |  |  |  | 14 | 46.8 | ± | 51.9 | 27.9 | 8 | - | 123 | 0.136 |  |
|  | **Fellutanine A** | 11 | 38.3 | ± | 20.8 | 38.4 | 23.7 | - | 53.1 |  | 32 | 42.6 | ± | 37.1 | 37.8 | 5.65 | - | 129 | 0.109 |  |
|  | **Ilicicolin A** | 11 | 2.44 | ± | 1.75 | 2.44 | 1.2 | - | 3.68 |  | 4 |  |  |  |  | 2.11 | | | 0.459 |  |
|  | **Ilicicolin B** | 47 | 6.01 | ± | 5.7 | 4.17 | 0.64 | - | 19.2 |  | 46 | 11.5 | ± | 18.6 | 2.61 | 0.53 | - | 62.1 | 0.843 |  |
|  | **Isofusidienol** |  |  |  |  |  |  |  |  |  | 7 | 10.7 | ± | 9.68 | 10.7 | 3.89 | - | 17.6 | 0.508 |  |
|  | **Mollicellin D** |  |  |  |  |  |  |  |  |  | 14 | 75.5 | ± | 118 | 25.2 | 1.56 | - | 250 | 0.137 |  |
|  | **Monocerin** | 47 | 12.56 | ± | 23.3 | 3.93 | 0.91 | - | 73.4 |  | 36 | 14.3 | ± | 17.6 | 7.35 | 0.64 | - | 52.9 | 0.488 |  |
|  | **Trichotetronine** |  |  |  |  |  |  |  |  |  | 4 |  | | |  | 1,530 | | | >0.999 |  |
| ***Penicillium* spp.** | **7-Hydroxypestalotin** |  |  |  |  |  |  |  |  |  | 21 | 6.76 | ± | 6.30 | 5.42 | 1.08 | - | 16.4 | 0.068 |  |
|  | **Andrastin A** | 84 | 1,030 | ± | 1,850 | 90.8 | 4.02 | - | 5,840 |  | 86 | 3,860 | ± | 4,160 | 2,170 | 19.6 | - | 13,100 | 0.03 | * |
|  | **Andrastin B** | 74 | 508 | ± | 718 | 140 | 6.96 | - | 2,270 |  | 79 | 3,670 | ± | 4,300 | 1,900 | 5.81 | - | 14,100 | 0.05 |  |
|  | **Andrastin C** | 84 | 9,580 | ± | 14,800 | 723 | 71.3 | - | 36,720 |  | 79 | 45,200 | ± | 58,100 | 32,800 | 21.5 | - | 252,000 | 0.179 |  |
|  | **Atpenin A5** |  |  |  |  |  |  |  |  |  | 11 | 15.6 | ± | 20.2 | 5.73 | 2.21 | - | 38.9 | 0.262 |  |
|  | **Chevalone C** | 47 | 11.2 | ± | 11.5 | 9.49 | 2.2 | - | 39.6 |  | 4 |  |  |  |  | 0.43 | | | <0.001 | * |
|  | **Citrinin** | 5 |  |  |  |  | 99.7 | | |  |  |  |  |  |  |  |  |  | 0.404 |  |
|  | **Eremofortin A** |  |  |  |  |  |  |  |  |  | 4 |  |  |  |  | 57 | | | >0.999 |  |
|  | **Marcfortine A** | 63 | 201 | ± | 531 | 16.7 | 4.11 | - | 1,880 |  | 68 | 2,027 | ± | 3,060 | 777 | 1.01 | - | 12,900 | 0.196 |  |
|  | **Mycophenolic acid** | 79 | 2,530 | ± | 2,740 | 1,960 | 18.1 | - | 7,450 |  | 82 | 5,570 | ± | 9,130 | 2,000 | 2.59 | - | 30,900 | 0.771 |  |
|  | **Mycophenolic acid IV** | 63 | 108 | ± | 155 | 57.1 | 1.57 | - | 570 |  | 68 | 199 | ± | 307 | 50.5 | 0.41 | - | 1,050 | 0.771 |  |
|  | **Pestalotin** |  |  |  |  |  |  |  |  |  | 43 | 7.13 | ± | 4.31 | 7.66 | 1.42 | - | 13.1 | <0.001 | * |
|  | **Questiomycin A** | 11 | 27.4 | ± | 29.6 | 27.4 | 6.46 | - | 48.3 |  | 64 | 27.3 | ± | 33.1 | 15.2 | 4.24 | - | 111 | <0.001 | * |
|  | **Quinolactacin A** |  |  |  |  |  |  |  |  |  | 7 | 0.06 | ± | 0.04 | 0.06 | 0.03 | - | 0.08 | 0.508 |  |
|  | **Roquefortine C** | 79 | 2,270 | ± | 2,940 | 1,150 | 64.5 | - | 10,900 |  | 86 | 6,360 | ± | 6,080 | 6,530 | 6.36 | - | 20,000 | 0.148 |  |
|  | **Roquefortine D** | 58 | 756 | ± | 1,340 | 160 | 32.7 | - | 4,400 |  | 50 | 6,220 | ± | 9,690 | 1,970 | 129 | - | 31,200 | 0.559 |  |

^1^Samples with values > limit of detection (LOD); ^2^Excluding data < LODIn case values >LOD and < limit of quantification (LOQ), LOQ/2 was used for calculation. * Significantly different (p-value < 0.05)

**Supplementary Table S2. Cont.** Occurrences and levels of mycotoxins and other metabolites detected in spots of mouldy grass and maize silages

|  |  | **Grass silage (n=19)** | | | | | | | |  | **Maize silage (n=28)** | | | | | | | | **Mann-Whitney Test** | |
| --- | --- | --- | --- | --- | --- | --- | --- | --- | --- | --- | --- | --- | --- | --- | --- | --- | --- | --- | --- | --- |
| **Group** | **Metabolite** | **Positive Samples (%)^1^** | **Concentration^2^** | | | | | | |  | **Positive Samples (%)^1^** | **Concentration^2^** | | | | | | | ***p*-value** | |
|  |  |  | **Average ± SD** | | | **Median** | **Range** | | |  |  | **Average ± SD** | | | **Median** | **Range** | | |  |  |
| **Unspecific metabolites** | **3-Nitropropionic acid** | 26 | 109 | ± | 206 | 17.2 | 4.78 | - | 478 |  | 54 | 21 | ± | 40 | 7.23 | 1.7 | - | 159 | 0.191 |  |
|  | **Asperglaucide** | 5 |  |  |  |  | 0.14 | | |  | 7 | 0.22 | ± | 0.04 | 0.22 | 0.19 | - | 0.25 | 0.705 |  |
|  | **Brevianamid F** | 74 | 60.97 | ± | 40.6 | 54.9 | 17.7 | - | 158 |  | 75 | 75.8 | ± | 123 | 39.5 | 9.02 | - | 593 | 0.791 |  |
|  | **Chlorocitreorosein** |  |  |  |  |  |  |  |  |  | 21 | 43.2 | ± | 61.7 | 14.1 | 0.89 | - | 160 | 0.068 |  |
|  | **Chrysophanol** | 26 | 457 | ± | 721 | 117 | 75.8 | - | 1,740 |  | 36 | 1,410 | ± | 2,510 | 167 | 29 | - | 7,180 | 0.546 |  |
|  | **Citreorosein** | 21 | 72.97 | ± | 64.5 | 73.7 | 12.6 | - | 132 |  | 50 | 212 | ± | 469 | 22.4 | 2.47 | - | 1,320 | 0.094 |  |
|  | **Cyclo(L-Pro-L-Tyr)** | 95 | 527 | ± | 330 | 479 | 59.9 | - | 1,240 |  | 100 | 259 | ± | 213 | 229 | 3.53 | - | 725 | 0.016 | * |
|  | **Cyclo(L-Pro-L-Val)** | 100 | 1,950 | ± | 1,510 | 1,750 | 17.6 | - | 4,940 |  | 93 | 923 | ± | 911 | 668 | 6.98 | - | 3,380 | 0.007 | * |
|  | **Emodin** | 95 | 167 | ± | 422 | 11.3 | 2.56 | - | 1,430 |  | 89 | 104 | ± | 165 | 47.7 | 0.48 | - | 712 | 0.602 |  |
|  | **Endocrocin** | 26 | 2,500 | ± | 3,000 | 897 | 159 | - | 7,230 |  | 71 | 1,940 | ± | 3,110 | 518 | 12.5 | - | 13,200 | 0.015 | * |
|  | **Fallacinol** | 16 | 5.81 | ± | 5.67 | 4.92 | 0.64 | - | 11.9 |  | 18 | 24.1 | ± | 34 | 4.63 | 0.47 | - | 81.1 | 0.849 |  |
|  | **Iso-Rhodoptilometrin** | 32 | 3.9 | ± | 3.29 | 2.88 | 0.95 | - | 10.1 |  | 54 | 8.62 | ± | 7.03 | 5.1 | 0.41 | - | 20.2 | 0.073 |  |
|  | **Lecanoic acid** | 11 | 3.0 | ± | 2.77 | 3.01 | 1.05 | - | 4.97 |  | 7 | 4.7 | ± | 4.5 | 4.7 | 1.53 | - | 7.86 | 0.94 |  |
|  | **Myriocin** |  |  |  |  |  |  |  |  |  | 11 | 2,330 | ± | 1,370 | 1,940 | 1,210 | - | 3,860 | 0.262 |  |
|  | **N-Benzoyl-Phenylalanine** | 5 |  |  |  |  | 5.32 | | |  | 11 | 3.72 | ± | 2.37 | 3.11 | 1.71 | - | 6.34 | 0.55 |  |
|  | **Rugulusovin** | 63 | 50.0 | ± | 32.6 | 41.55 | 8.78 | - | 135 |  | 68 | 44.4 | ± | 33.1 | 39.8 | 4.73 | - | 122 | 0.96 |  |
|  | **Skyrin** | 42 | 19.9 | ± | 34.8 | 4.94 | 0.61 | - | 100 |  | 39 | 45.6 | ± | 87.5 | 15.9 | 1.03 | - | 301 | 0.901 |  |
|  | **Tryptophol** | 89 | 773 | ± | 860 | 466 | 66.7 | - | 2,900 |  | 96 | 527 | ± | 491 | 350 | 39.1 | - | 1,900 | 0.66 |  |
|  | **Usnic acid** | 16 | 2.1 | ± | 0.64 | 2.07 | 1.47 | - | 2.75 |  | 14 | 1.66 | ± | 1.18 | 1.29 | 0.69 | - | 3.37 | 0.748 |  |

^1^Samples with values > limit of detection (LOD); ^2^Excluding data < LOD. In case values >LOD and < limit of quantification (LOQ), LOQ/2 was used for calculation.

* Significantly different (p-value < 0.05)

**Supplementary Table S3.** Significative differences in the levels of mycotoxins and other metabolites detected in spots of mouldy grass and maize silages during year 2019 and 2020

|  |  | **Grass silage** | | | | | | | | | |  | **Maize silage** | | | | | | | | | |
| --- | --- | --- | --- | --- | --- | --- | --- | --- | --- | --- | --- | --- | --- | --- | --- | --- | --- | --- | --- | --- | --- | --- |
|  |  | **2019 (n=9)** | | |  | **2020 (n=10)** | | |  | **Mann-Whitney Test** | |  | **2019 (n=18)** | | |  | **2020 (n=10)** | | |  | **Mann-Whitney Test** | |
|  |  |  | **Concentration** | |  |  | **Concentration** | |  |  |  |  |  | **Concentration** | |  |  | **Concentration** | |  |  |  |
| **Metabolite /**  **Group** |  | **Positive samples (n)** | **Average** | **Median** |  | **Positive samples (n)** | **Average** | **Median** |  | **P-value** |  |  | **Positive samples (n)** | **Average** | **Median** |  | **Positive samples (n)** | **Average** | **Median** |  | **P-value** |  |
| Andrastin C |  | 7 | 384 | 398 |  | 9 | 12,600 | 4,270 |  | 0.006 | * |  | 14 | 31,928 | 38,052 |  | 8 | 61,810 | 28,930 |  | 0.161 |  |
| Apicidin |  | 0 |  |  |  | 1 | 7.92 | 7.92 |  | >0.999 | ns |  | 15 | 24.3 | 20.0 |  | 5 | 22.2 | 9.88 |  | 0.039 | * |
| Atpenin A5 |  | 0 |  |  |  | 0 |  |  |  | >0.999 | ns |  | 2 | 20.5 | 20.5 |  | 1 | 5.73 | 5.73 |  | 0.767 | ns |
| Beauvericin |  | 7 | 24.9 | 3.77 |  | 2 | 1.61 | 1.61 |  | 0.016 | * |  | 17 | 38.2 | 23.8 |  | 7 | 10.5 | 7.74 |  | 0.007 | * |
| Brevianamid F |  | 9 | 47.7 | 47.8 |  | 5 | 84.9 | 104 |  | 0.273 | ns |  | 17 | 86.5 | 44.4 |  | 4 | 30.3 | 23.8 |  | 0.001 | * |
| Chrysogine |  | 7 | 40.0 | 19.8 |  | 3 | 21.4 | 26.5 |  | 0.041 | * |  | 3 | 7.49 | 4.30 |  | 5 | 6.13 | 4.57 |  | 0.058 | ns |
| Citreorosein |  | 3 | 53.3 | 22.0 |  | 1 | 132 | 132 |  | 0.443 | ns |  | 13 | 226 | 17.8 |  | 1 | 27.0 | 27.0 |  | 0.003 | * |
| Culmorin |  | 4 | 69.2 | 46.1 |  | 4 | 98.4 | 102 |  | >0.999 | ns |  | 12 | 231 | 118 |  | 10 | 388 | 241 |  | 0.001 | * |
| cyclo(L-Pro-L-Tyr) |  | 8 | 497 | 500 |  | 10 | 551 | 479 |  | 0.447 | ns |  | 18 | 327 | 296 |  | 10 | 139 | 85.3 |  | 0.024 | * |
| Deoxynivalenol |  | 1 | 9.24 | 9.24 |  | 2 | 24.8 | 24.8 |  | 0.598 | ns |  | 12 | 205 | 191 |  | 10 | 393 | 276 |  | 0.026 | * |
| Emodin |  | 8 | 178 | 11.3 |  | 10 | 159 | 11.3 |  | 0.720 | ns |  | 17 | 141 | 102 |  | 8 | 23.7 | 10.2 |  | 0.018 | * |
| Endocrocin |  | 5 | 2,500 | 897 |  | 0 |  |  |  | 0.011 | * |  | 16 | 2,099 | 518 |  | 4 | 1,300 | 1,060 |  | 0.014 | * |
| Enniatin A |  | 7 | 1.36 | 0.81 |  | 0 |  |  |  | 0.001 | * |  | 11 | 0.77 | 0.54 |  | 1 | 1.66 | 1.66 |  | 0.023 | * |
| Enniatin A1 |  | 9 | 4.35 | 2.20 |  | 2 | 1.84 | 1.84 |  | 0.001 | * |  | 17 | 12.1 | 4.98 |  | 4 | 5.50 | 3.03 |  | 0.007 | * |
| Enniatin B1 |  | 9 | 15.3 | 8.93 |  | 4 | 6.40 | 4.83 |  | 0.014 | * |  | 13 | 23.8 | 7.49 |  | 6 | 9.7 | 3.42 |  | 0.257 | ns |
| Enniatin B2 |  | 5 | 0.70 | 0.44 |  | 0 |  |  |  | 0.011 | * |  | 8 | 0.38 | 0.34 |  | 1 | 1.06 | 1.06 |  | 0.139 | ns |
| Eremofortin A |  | 0 |  |  |  | 0 |  |  |  | >0.999 | ns |  | 1 | 57.0 | 57.0 |  | 0 |  |  |  | >0.999 | ns |
| Kojic acid |  | 4 | 63.2 | 43.5 |  | 0 |  |  |  | 0.033 | * |  | 12 | 97.7 | 54.3 |  | 0 |  |  |  | 0.001 | * |

^1^Samples with values > limit of detection (LOD); ^2^Excluding data < LODIn case values >LOD and < limit of quantification (LOQ), LOQ/2 was used for calculation.

* Significantly different (p-value < 0.05)

**Supplementary Figure S1.** Co-occurrence (%) of fungal species isolated from mouldy spots of (a) grass and (b) maize silage.

**
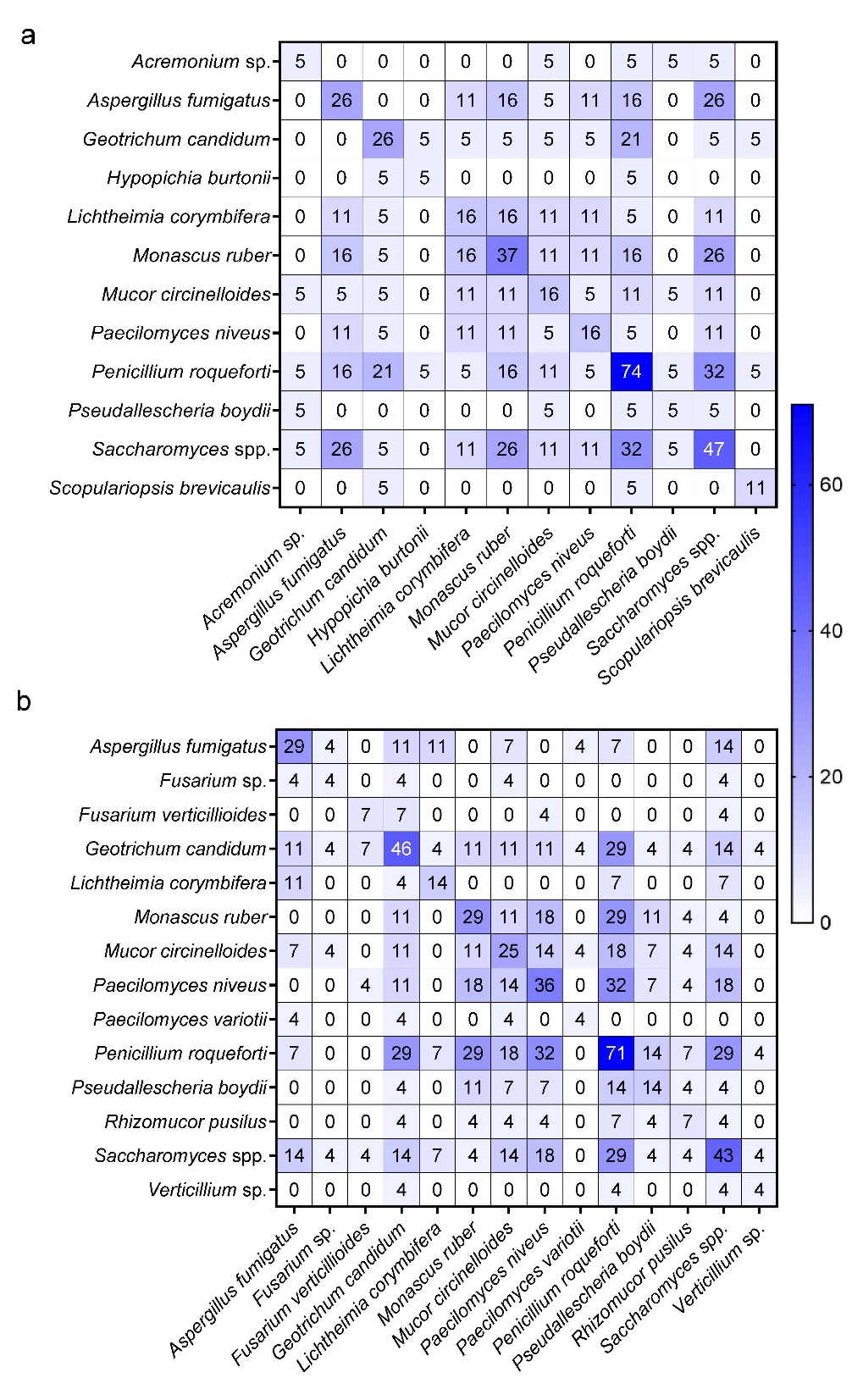
**

**Supplementary Figure S2.** Linear regressions and bar diagram showing significant associations detected in the analysed mouldy silages. (a) Association between levels of the total as well as (b) of andrastin A (AND A), andrastin B (AND B), andrastin C (AND C) along with roquefortine C (ROC C) and roquefortine D (ROC D) with concentration total fungal metabolites detected in mouldy spots of grass silage. (c) Relationship between counts of *P. roqueforti* and levels of total *Penicillium* metabolites. (d) Associations between total *Penicillium* metabolites and specific metabolites (AND A, AND B and ROC D) in mouldy maize silages. (e) Relationship between *Aspergillus-*derived metabolites and unspecific metabolites in mouldy spots of maize silage. (f) Relationship between zearalenone and deoxynivalenol in mouldy spots of maize. (g) Correlation of festuclavine with the *Penicillium*-derived metabolites (AND A, ROQ C and ROC D) in mouldy grass silage. Sy.x = Standard error of estimate. Significance level at *p*-value < 0.05).

**
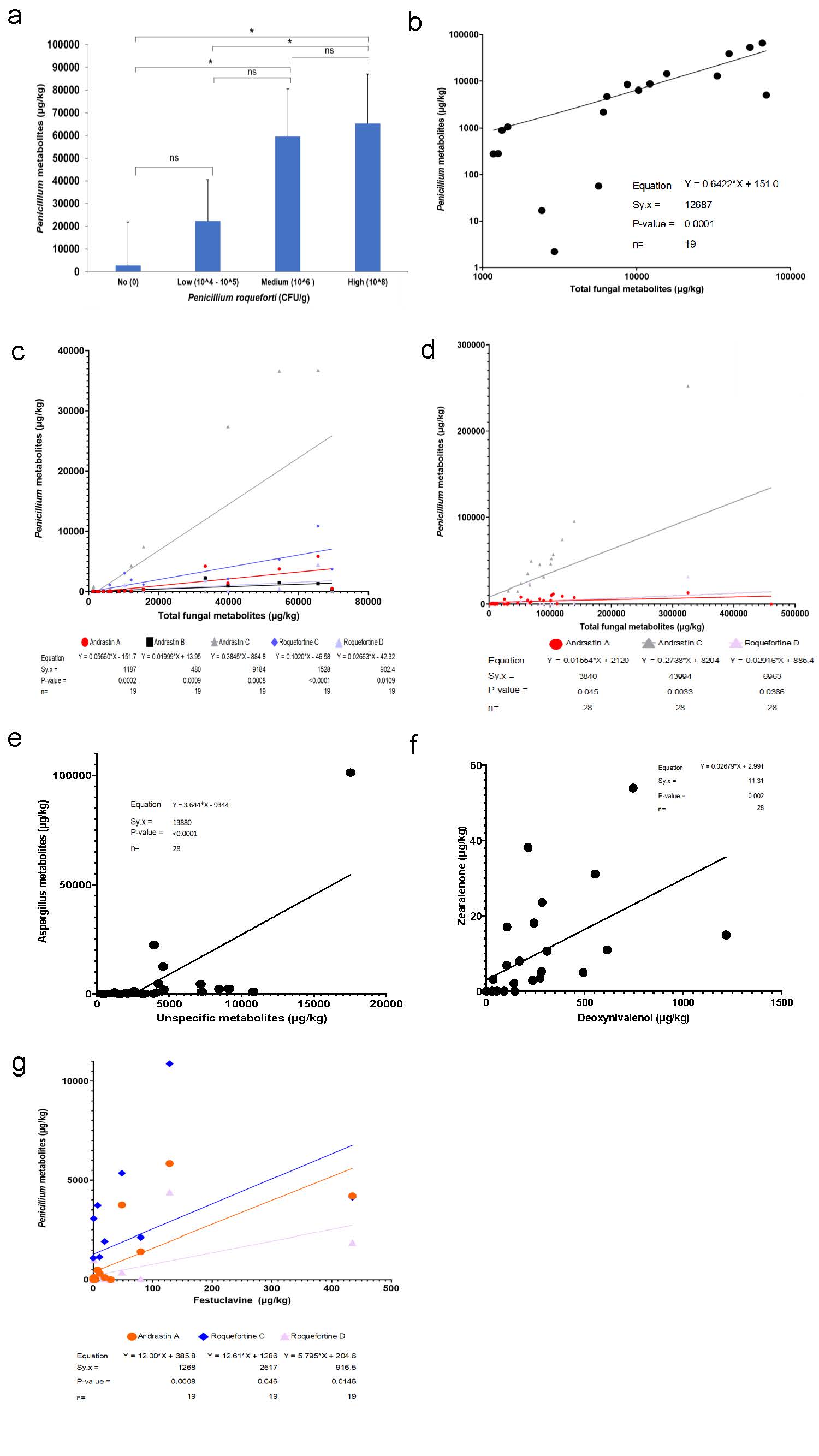
**

**Supplementary Figure S2. Cont.**

**
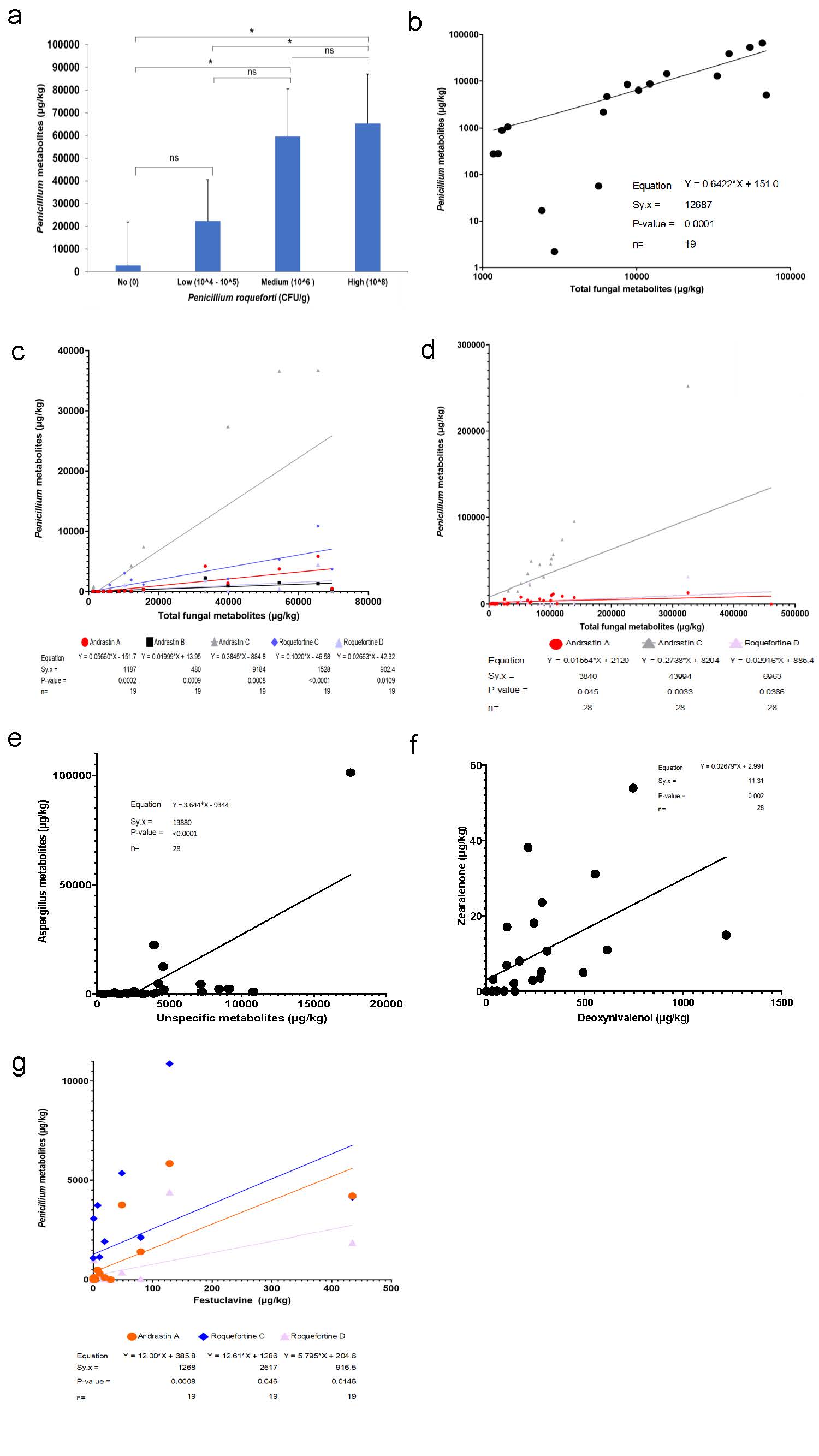
**
